# MST1R-targeted therapy in the battle against gallbladder cancer

**DOI:** 10.1186/s13578-024-01290-w

**Published:** 2024-08-29

**Authors:** Wei Wang, Chao Huang, Li Zhang, Liqin Yu, Yangming Liu, Puxiongzhi Wang, Rongmu Xia

**Affiliations:** 1 Department of Hepatobiliary and Pancreatic Surgery, Shanghai Sixth People’s Hospital Affiliated to Shanghai Jiao Tong University School of Medicine, No. 600 Yishan Road, Shanghai, 200233 China; 2 Department of Cell Biology, Medical School, Kunming University of Science and Technology, Kunming, 650500 China; 3 Department of Pathology, Shanghai Sixth People’s Hospital Affiliated to Shanghai Jiao Tong University School of Medicine, Shanghai, 200233 China; 4 Department of Gastroenterology, The Second Affiliated Hospital of Fujian, University of Traditional Chinese Medicine, Fuzhou, 350003 China

**Keywords:** Combination therapy, Gallbladder cancer, JMJD6, MST1R, Targeted therapy

## Abstract

**Background:**

Gallbladder cancer (GBC) is characterized by high mortality rate. Our study sought therapeutic candidates for GBC.

**Results:**

Bioinformatics analysis identified significant upregulation of MST1R in GBC. In vitro experiments demonstrated that the MST1R inhibitor MGCD-265 effectively restrained GBC cell proliferation at lower concentrations. Additionally, it induced cycle arrest and apoptosis in GBC cells in a dose-dependent manner. Mouse models exhibited that MGCD-265 treatment significantly diminished the proliferative capacity of GBC-SD cells. Transcriptomics sequencing revealed significant transcriptome alterations, with 200 transcripts upregulated and 883 downregulated. KEGG and GO analyses highlighted enrichment in processes like cell adhesion and pathways such as protein digestion and absorption. Downstream genes analysis identified JMJD6 upregulation post-MGCD-265 treatment. In vivo experiments confirmed that combining MGCD-265 with the JMJD6 inhibitor SKLB325 enhanced the anticancer effect against GBC.

**Conclusion:**

Overall, targeting MST1R and its downstream genes, particularly combining MGCD-265 with SKLB325, holds promise as a therapeutic strategy for GBC.

**Supplementary Information:**

The online version contains supplementary material available at 10.1186/s13578-024-01290-w.

## Background

Gallbladder cancer (GBC), ranking as the sixth most common gastrointestinal tract tumor [1, 2], with an overall 5-year survival rate below 5% [3]. The majority of patients receive their diagnosis at an advanced stage, with less than 20% qualifying for potentially curative surgical resection [4, 5]. While targeted therapy has been a pivotal area of research and development in tumor therapy, its application to gallbladder cancer remains in the initial stages of exploration [1]. Extant pharmaceutical approaches to biliary tract neoplasms are constrained, marked by suboptimal efficacy. Consequently, there exists a pressing imperative to discern viable targets and pharmacotherapeutic agents with heightened effectiveness in addressing this medical challenge.

A comprehensive understanding of the precise causes and pathogenesis of gallbladder cancer has yet to be achieved. Fewer drug targets are available for GBC to date. Recent research has made progress in this area by identifying potential genetic variants that are linked to the development of gallbladder cancer through association studies involving single nucleotide polymorphisms (SNPs). Promising candidates for therapeutic intervention in gallbladder cancer have been identified, including the ABCG8 and TRAF3 genes, which exhibit noteworthy correlations with the disease [6]. Furthermore, genetic variations associated with inflammation, apoptosis, DNA repair, drug metabolism, hedgehog signaling, Wnt signaling, TGF-β, MET-related pathways, or aberrant levels of associated proteins have demonstrated significant associations with gallbladder cancer [7–9]. The aforementioned insights into genetic variation provide significant understanding of the pathogenesis of GBC and contribute to the elucidation of precise prevention and targeted therapy strategies. This knowledge plays a crucial role in the advancement of tailored drug development for gallbladder cancer. Using the Hedgehog signaling pathway as an illustrative example, it is noteworthy that although this pathway is typically inactive in adults, mutations or reactivations of associated genes can have a profound impact on tumor development [10–12]. Notably, the Hedgehog pathway has seen targeted inhibitors like Erivedge (vismodegib) and Odomzo (sonidegib) approved by the US FDA for basal cell carcinoma treatment [13, 14]. The pursuit of novel small molecule targeted drugs for gallbladder cancer holds promise in broadening therapeutic options for patients and enhancing the clinical efficacy in treating this malignancy.

The exploration for small molecule chemotherapeutic agents targeting gallbladder cancer holds promise in diversifying therapeutic options and enhancing the clinical efficacy of gallbladder cancer treatment. In this study, we observed that MGCD-265, a small molecule inhibitor targeting Recepteur d’Origine Nantais (MST1R, RON), exhibited a low IC_50_ value for gallbladder cancer cell lines. Moreover, MGCD-265 treatment induced gallbladder cancer cell cycle arrest and apoptosis. To further investigate the potential mechanisms underlying the inhibitory effect of MGCD-265 on gallbladder cancer cell proliferation, we conducted a series of in vivo and in vitro experiments, in conjunction with transcriptomic sequencing. These investigations provided preliminary confirmation of MGCD-265’s anti-gallbladder cancer effect and guided the screening of small molecule inhibitors for potential co-administration, aiming for a more effective approach to combating gallbladder cancer.

## Materials and methods

### Cell culture

Human gallbladder cancer cell lines GBC-SD, NOZ, and SGC-996 were obtained from the Shanghai Cell Bank of the Chinese Academy of Sciences. These cell lines were cultured in a mixture consisting of 89% RPMI 1640 medium (Gibco), 10% fetal bovine serum (Gibco), and 1% penicillin/ /streptomycin (Gibco), respectively. The cells were maintained at a temperature of 37 °C in a cell culture incubator (Thermo Fisher Scientific Inc.) with 5% CO_2_ and saturated humidity. For subsequent experiments, tumor cells in the logarithmic growth phase were utilized.

### Western blot

GBC-SD cells were exposed to varying concentrations of MGCD-265 (#S1361, Selleck) for 24 h. RIPA lysate (#PC101, Epizyme Biotech, Shanghai, China), supplemented with Protease Inhibitor Cocktail (#P8340, Sigma-Aldrich LLC), was employed for cell lysis, followed by a 30-minute incubation on ice and subsequent centrifugation at 4 °C for 15 min at 12,000 g. The resulting supernatant was collected and assessed for protein concentration using the BCA method (#P0012, Beyotime Biotechnology, China). Gel electrophoresis was executed utilizing an SDS-PAGE separator gel (#PG112 and #PG113, Epizyme Biotech, Shanghai, China), with each sample loaded containing an equal protein quantity of 30 µg. Subsequently, proteins were electrophoretically separated and transferred to a PVDF membrane (#IPVH00010, Millipore, Sigma-Aldrich LLC). These membranes were then blocked using a 5% skimmed milk solution (#A600669-0250, Sangon Biotech (Shanghai) Co., Ltd., China) at room temperature for 2 h. Thereafter, the membranes were horizontally cut to probe proteins with different molecular weights. Overnight incubation at 4 °C with the respective primary antibodies was carried out, followed by a triple wash with TBS/Tween Buffer (PS103, Epizyme Biotech, Shanghai, China) and subsequent co-incubation with the secondary antibody at room temperature for 2 h. Immunoblots were tested using an BeyoECL Plus Kit (#P0018S, Beyotime Biotechnology, China), and protein quantification was conducted utilizing Image J software. GAPDH and β-actin were employed as the internal reference for normalization.

The antibodies used in this study are listed below. Anti-p21 (Santa Cruz Biotechnology, #sc-6246, working dilution 1:2000), anti-HSP90AA1 (Abcam, ab303516l, working dilution 1:500), anti-Cyclin D1 (Abcam, ab226977, working dilution 1:2000), anti-Cyclin B1 (Abcam, ab181593, working dilution 1:2000), anti-CDK4 (Abcam, ab137675, working dilution 1:2000), anti-JMJD6 (Abcam, ab307654, working dilution 1:500), anti-CDC25A (Abcam, ab2357, working dilution 1:500), anti-SLC1A5 (Abcam, ab237704, working dilution 1:500), anti-SLC7A11 (Abcam, ab307602, working dilution 1:500), anti-GAPDH (Abcam, ab8227, working dilution 1:5000), anti-Cleaved-caspase3 (Cell Signaling Technology, #9664, working dilution 1:1500), anti-Caspase3 (Cell Signaling Technology, #9662, working dilution 1:1500), anti-Cleaved-PARP (Cell Signaling Technology, #5625, working dilution 1:1500), anti-β-actin (Cell Signaling Technology, #4970, working dilution 1:5000), Goat Anti-Rabbit IgG H&L (HRP) secondary antibody (Abcam, ab6721, working dilution 1:8000), and Goat Anti-Mouse IgG H&L (HRP) secondary antibody (Abcam, ab205719, working dilution 1:8000).

### RNA extraction, reverse transcription and quantitative real-time polymerase chain reaction (RT-qPCR) analysis

Following drug treatment, cancer cells underwent a pre-cooled PBS (Gibco) wash, and total RNA extraction from the cells was executed using TRIzol™ LS Reagent (#10296010, Invitrogen, Thermo Fisher Scientific Inc.). Next, the RNA concentration was assessed utilizing a NanoDrop spectrophotometer (NanoDrop Technologies Inc.). Reverse transcription of RNA into cDNA was carried out HiScript II Q Select RT SuperMix for qPCR (+ gDNA wiper) according to manufacturer instructions (#R233-01, Nanjing Vazyme Biotechnology Co., LTD, China). RT-qPCR was conducted employing a LightCycler 96 fluorescent quantitative PCR instrument using 2×AceQ qPCR SYBR Green Master Mix (#Q111-02, Nanjing Vazyme Biotechnology Co., LTD, China). The expression of β-actin or GAPDH served as the internal control. The relative transcript levels of genes were statistically analyzed using the 2^−ΔΔCT^ method.

The primers used are listed below. *AKT2* forward: 5’-ACCACAGTCATCGA GAGGACC-3’, reverse: 5’-GGAGCCACACTTGTAGTCCA-3’; *AKT3* forward: 5’-TGAAGTGGCACACACTCTAACT-3’, reverse: 5’-CCGCTCTCTCGACAAATG GA-3’; *CDK2AP1* forward: 5’-ATGTCTTACAAACCGAACTTGGC-3’, reverse: 5’-GCCCGTAGTCACTGAGCAG-3’; *CDKN1A* forward: 5’-CGATGGAACTTCGACT TTGTCA-3’, reverse: 5’-GCACAAGGGTACAAGACAGTG-3’; *MAP2K6* forward: 5’-GAAGCATTTGAACAACCTCAGAC-3’, reverse: 5’-CCTGGCTATTTACTGT GGCTC-3’; *MMP14* forward: 5’-GGCTACAGCAATATGGCTACC-3’, reverse: 5’-GATGGCCGCTGAGAGTGAC-3’; *MMP2* forward: 5’-TACAGGATCATTGGCTA CACACC-3’, reverse: 5’-GGTCACATCGCTCCAGACT-3’; *CDK4* forward: 5’-CTGGTGTTTGAGCATGTAGACC-3’, reverse: 5’-GATCCTTGATCGTTTCGGC TG-3’; *MMP24* forward: 5’-GCCGGGCAGAACTGGTTAAA-3’, reverse: 5’-CCCGTAAAACTGCTGCATAGT-3’; *MMP26* forward: 5’-TCGGAATGGGACAG ACCTACT-3’, reverse: 5’-TCAAAGGGGTCACATTGCTCC-3’; *MMP7* forward: 5’-GAGTGAGCTACAGTGGGAACA-3’, reverse: 5’-CTATGACGCGGGAGTTTAA CAT-3’; *PIK3AP1* forward: 5’-GAGCCAGAGACCTACGTGG-3’, reverse: 5’-TGTCATCCAGCTTACATCTCACA-3’; *PIK3R1* forward: 5’-ACCACTACCGGA ATGAATCTCT-3’, reverse: 5’-GGGATGTGCGGGTATATTCTTC-3’; *PIK3CG* forward: 5’-GGCGAAACGCCCATCAAAAA-3’, reverse: 5’-GACTCCCGTGC AGTCATCC-3’; *PIK3CD* forward: 5’-AAGGAGGAGAATCAGAGCGTT-3’, reverse: 5’-GAAGAGCGGCTCATACTGGG-3’; *PIK3CB* forward: 5’-TATTTGGACTT TGCGACAAGACT-3’, reverse: 5’-TCGAACGTACTGGTCTGGATAG-3’; *PIK3C2B* forward: 5’-TCAGGGCAATGGGGAACAC-3’, reverse: 5’-CGTAACAGCTTGA GGTCGGTC-3’; *ODC1* forward: 5’-TTTACTGCCAAGGACATTCTGG-3’, reverse: 5’-GGAGAGCTTTTAACCACCTCAG-3’; *CACYBP* forward: 5’-CTCCCATTAC AACGGGCTATAC-3’, reverse: 5’-GAACTGCCTTCCACAGAGATG-3’; *HSPA6* forward: 5’-CAAGGTGCGCGTATGCTAC-3’, reverse: 5’-GCTCATTGATGATCC GCAACAC-3’; *AHSA1* forward: 5’-ACGCCACCAACGTCAACAA-3’, reverse: 5’-ACAGTGTTTTCAGCTTATCCGTG-3’; *DNAJA1* forward: 5’-AGGAGCAGTAGA GTGCTGTCC-3’, reverse: 5’-TCTCGAACTATCTTCCTTCCGT-3’; *BAG3* forward: 5’-TGGGAGATCAAGATCGACCC-3’, reverse: 5’-GGGCCATTGGCAGAGGATG-3’; *CHORDC1* forward: 5’-CCTTGCTGTGCTACAACCG-3’, reverse: 5’-CGGAA CACCTGGGTGGTATG-3’; *STIP1* forward: 5’-CCTTACAGTGCTACTCCGA AGC-3’, reverse: 5’-ATAGGCAGCAGAACGGTTGC-3’; *PSAT1* forward: 5’-TGCCGCACTCAGTGTTGTTAG-3’, reverse: 5’-GCAATTCCCGCACAAGATTCT-3’; *HSPD1* forward: 5’-ATGCTTCGGTTACCCACAGTC-3’, reverse: 5’-AGCCCGAGTGAGATGAGGAG-3’; *SLC7A11* forward: 5’-TCTCCAAAGGAGG TTACCTGC-3’, reverse: 5’-AGACTCCCCTCAGTAAAGTGAC-3’; *HSPA8* forward: 5’-ACCTACTCTTGTGTGGGTGTT-3’, reverse: 5’-GACATAGCTTGGAGTGGT TCG-3’; *HSP90AA1* forward: 5’-AGGAGGTTGAGACGTTCGC-3’, reverse: 5’-AGAGTTCGATCTTGTTTGTTCGG-3’; *HSPH1* forward: 5’-ACAGCCATG TTGTTGACTAAGC-3’, reverse: 5’-GCATCTAACACAGATCGCCTCT-3’; *DNAJB1* forward: 5’-AAGGCATGGACATTGATGACC-3’, reverse: 5’-GGCCAAAGTTCA CGTTGGT-3’; *HSPA1B* forward: 5’-TTTGAGGGCATCGACTTCTACA-3’, reverse: 5’-CCAGGACCAGGTCGTGAATC-3’; *HSPA1A* forward: 5’-GCCTTTCCAAGA TTGCTGTT-3’, reverse: 5’-TCAACATTGCAAACACAGGA-3’; *FKBP4* forward: 5’-GAAGGCGTGCTGAAGGTCAT-3’, reverse: 5’-TGCCATCTAATAGCCAGCCAG-3’; *MTHFD2* forward: 5’-CTGCGACTTCTCTAATGTCTGC-3’, reverse: 5’-CTCGCCAACCAGGATCACA-3’; *MRPL18* forward: 5’-GCAGCGAAACCTGAA GTGGA-3’, reverse: 5’-GTGCCAGAACTCACGGGAG-3’; *SLC1A5* forward: 5’-GAGCTGCTTATCCGCTTCTTC-3’, reverse: 5’-GGGGCGTACCACATGATCC-3’; *CDC25A* forward: 5’-GTGAAGGCGCTATTTGGCG-3’, reverse: 5’-TGGTTGCTCATAATCACTGCC-3’; *ZFAND2A* forward: 5’-GATCATTTTCCATA CGCTGCAC-3’, reverse: 5’-CGTCTGGTATCTGGCCCTTTT-3’; *RASSF1* forward: 5’-AGGACGGTTCTTACACAGGCT-3’, reverse: 5’-TGGGCAGGTAAAA GGAAGTGC-3’; *JMJD6* forward: 5’-TTGGACCCGGCACAACTACTA-3’, reverse: 5’-TCTGCCCTTTCCACGTTATCC-3’; *MICB* forward: 5’-TCTTCGTTACAACC TCATGGTG-3’, reverse: 5’-TCCCAGGTCTTAGCTCCCAG-3’; *DNAJB4* forward: 5’-GCAGGAGGTACTGATGGACAA-3’, reverse: 5’-ACCACCCATTCGTCTT CCAAA-3’; *DDIAS* forward: 5’-AGGTTCAGATGCCAGTAACTTCT-3’, reverse: 5’-AGTGATTGTTAGGTGCCTGAGA-3’; *BYSL* forward: 5’-GGCTGAGCCGAC GGATTTT-3’, reverse: 5’-CCTCGTCATCTGATCCATCCTG-3’; *HSPA4L* forward: 5’-CGGCTTTCTCAACTGCTACAT-3’, reverse: 5’-ACCTGTCGCTGTACTCATT GG-3’; *GAPDH* forward: 5’-CAATGACCCCTTCATTGACC-3’, reverse: 5’-TGGAAGATGGTGATGGGATT-3’; *β-actin* forward: 5’-CCTCGCCTTTGCCGATCC-3’, reverse: 5’-GGATCTTCATGAGGTAGTCAGTC-3’.

### Animal experiments

Female BALB/c nude mice (6 weeks old, weighing 16–18 g) were housed at constant temperature (23 ± 2 °C) and controlled light (12 h light:12 h dark) under pathogen-free conditions. All experimental procedures strictly adhered to the applicable guidelines and regulations regarding animal research. The animal study protocol was approved by the Animal Care and Use Committee of Shanghai Sixth People’s Hospital Affiliated to Shanghai Jiao Tong University.

### Subcutaneous xenograft model

GBC-SD cells in the logarithmic growth phase were suspended and subcutaneously inoculated into the right hind limb of BALB/c nude mice in a 100 µL sterile PBS, containing 2 × 10^6^ cells. Upon the tumor volume reaching approximately 100 mm^3^, the mice were randomly assigned to one of three groups: a control group, a low-dose MGCD-265 group (5 mg/kg), and a high-dose MGCD-265 group (10 mg/kg). MGCD-265 was obtained from Selleck (#S1361). The drug was orally administered via gavage daily for 12 consecutive days, with a dosage of 100 µL per dose. Tumor size and body weight were measured at three-day intervals. On day 28 post-treatment, the mice were humanely euthanized using the cervical dislocation method, and the tumors were harvested immediately for subsequent experiments.

### Hematoxylin and eosin (H&E) staining

Fresh tissues underwent fixation using a 4% paraformaldehyde solution (#P0099, Beyotime Biotechnology) for 24 h. Subsequently, they were dehydrated through a series of graded ethanol concentrations and then embedded in paraffin. These Sect. (6 μm in thickness) were subjected to H&E staining (#C0105S, Beyotime Biotechnology), enabling the observation of pathological structures under light microscopy (Olympus, Japan).

### Immunohistochemistry (IHC)

Paraffin sections were subjected to dewaxing and rehydration processes, followed by immersion in Citrate Antigen Retrieval Solution (#P0081, Beyotime Biotechnology) for 10 min to facilitate antigen repair. Next, to inhibit endogenous peroxidase activity, a 3% hydrogen peroxide solution was applied, and the sections were washed with PBS three times. Blocking of non-specific binding sites was achieved through a 30-minute incubation with a 3% bovine serum albumin solution (#A610903, Sangon Biotech (Shanghai) Co., Ltd., China). Subsequently, the primary antibodies: anti-Cleaved-caspase3 (Cell Signaling Technology, #9664, working dilution 1:200), anti-CDK4 (Abcam, ab137675, working dilution 1:200), anti-CDK6 (Proteintech Group, Inc., #14052-1-AP, working dilution 1:200), anti-JMJD6 (Proteintech Group, Inc., 16476-1-AP, working dilution 1:200), anti-CDC25A (Abcam, ab2357, working dilution 1:100), anti-SLC1A5 (Abcam, ab237704, working dilution 1:100), and anti-SLC7A11 (Abcam, ab307602, working dilution 1:100) were incubated overnight at 4 °C. After washing with PBS, the sections were incubated with Goat Anti-Rabbit IgG H&L (HRP) secondary antibody (Abcam, ab6721, working dilution 1:1000), or Goat Anti-Mouse IgG H&L (HRP) secondary antibody (Abcam, ab205719, working dilution 1:1000) at room temperature for 1 h. Color reaction was performed using Horseradish catalase DAB color kit (#C520017, Sangon Biotech (Shanghai) Co., Ltd.). The sections were counterstained with hematoxylin, followed by a process of dehydration, sealing, and subsequent observation under a light microscope (Olympus, Japan).

### Total RNA isolation, library preparation and RNA transcriptomics sequencing

Tumor cells were cultivated in six-well plates and subjected to treatment with MGCD-265 for 24 h. Total RNA was then extracted from these cells utilizing TRIzol™ LS Reagent (#10296010, Invitrogen, Thermo Fisher Scientific Inc.) as per the manufacturer’s guidelines. The quality of the extracted total RNA was measured using Q9000 Micro-Volune Spectrophotometer (Quawell Ltd.), and the purity of the total RNA samples was assessed based on the ratio of absorbance at 260 nm and 280 nm, with ratios between 1.8 and 2.0 being considered acceptable. The RNA integrity numbers ≥ 7 was used for library preparation.

For library preparation, we employed the TruSeq^®^ Stranded mRNA Library Prep Kit (Illumina, USA) following manufacturer’s instructions. Subsequently, these libraries were utilized for paired-end sequencing, accomplished through the HiSeq X Sequencing Platform (Illumina, USA). To evaluate gene expression levels, RPKM values (Reads Per Kilobase Million) of transcripts and the ratio of transcripts were utilized to calculate the total RPKM values for each gene.

### Gene Ontology (GO) and Kyoto Encyclopedia Of Genes And Genomes (KEGG) Analysis

The utilization of GO functional annotation analysis and KEGG analysis is a prevalent approach in conducting extensive investigations on gene functional enrichment, encompassing analyses of biological process (BP), molecular function (MF), and cellular component (CC). KEGG databases serve as valuable resources for examining pertinent genomic data, biological pathways, diseases, and drugs. Pathway-based enrichment of genes is performed, and differential expressed genes (DEGs) are subjected to GO and KEGG analyses utilizing the “cluster profiler” tool within the R package.

### Drug synergy calculation

Following the drug intervention tests outlined above, the rate of inhibition was computed using the formula: rate of inhibition = ((1 - OD of experimental group/OD of control group)) x 100%. The half inhibitory concentration (IC_50_) was determined utilizing Origin Pro 50.7 software (OriginLab, Massachusetts, USA). To evaluate the synergistic effect of MGCD-265 and SKLB325, Q values were calculated based on King’s formula analysis: Q = E(A + B)/(E A + E B -E A XE B), where E(A + B) represents the combined drug inhibition rate, and EA and EB represent the inhibition rates of drugs A and B, respectively. A Q value of 0.85–1.15 indicates a simple summation of the effects of the two drugs, Q greater than 1.15 indicates enhancement (or synergy), and Q less than 0.85 indicates antagonism.

### GEO database-based analysis

The gallbladder cancer transcriptome dataset (GSE74948) was obtained from the NCBI GEO database, encompassing six samples: three cancerous tissue samples and three normal tissue samples. Corresponding platform annotation files were also acquired to convert probes into gene symbols. In cases where multiple probes corresponded to the same gene symbol, the average value was computed as the gene’s expression value. Differential expression analysis was carried out using the limma package, employing linear regression and empirical Bayesian methods. This analysis yielded the respective P-values and logFC values for the genes. The threshold for identifying differentially expressed genes was set at P-value < 0.05 and |logFC| > 2.

To further characterize these differentially expressed genes, the clusterProfiler package was utilized for GO and KEGG enrichment analysis. This analysis provided insights into the biological processes and pathways associated with the genes. Both up- and down-regulated genes underwent GO and KEGG analyses to comprehensively understand the alterations in molecular pathways. Additionally, exploring protein-protein interactions (PPIs) among the differentially expressed genes was crucial. Human protein-protein interaction data were obtained from the online STRING database, utilizing a minimum interaction score of 0.9 as the parameter value, thus ensuring high-confidence interactions were considered.

### Protein-protein interaction network

The STRING database is a valuable tool for both identifying known proteins and predicting protein interactions. In our study, we leveraged the capabilities of the STRING database to pinpoint differentially expressed genes (DEGs) that achieved a combined score surpassing 400. These identified DEGs were employed to construct mRNA-associated PPIs, which were further presented visually using Cytoscape (version 3.6.1).

### Analysis of the drug gene interaction database (DGIdb)

The DGIdb, a database focused on drug-gene interactions, offers comprehensive information regarding the correlation between genes and their established or potential pharmaceutical agents. In order to identify potential pharmaceutical candidates or small molecule inhibitors for gallbladder cancer, the DGIdb database was utilized to screen for such compounds, employing key target genes as screening criteria.

### CCK-8 assay

In the logarithmic growth phase, GBC-SD, NOZ, and SGC-996 cells were seeded in 96-well plates at a density of 2 × 10^3^ cells/well (100 µL/well). After cell attachment, the culture medium was replaced with fresh medium containing varying drug concentrations. Each drug concentration was tested in five replicate wells, with a corresponding set of blank control wells. The incubation continued for 24 h. Post-incubation, the culture medium was replaced with 100 µL of a CCK-8 (#B34304, Selleck) mixed with culture medium. Following a 4-hour incubation in the incubator, absorbance at 450 nm was measured using TECAN F50 Microplate Reader, allowing for the calculation of cell viability and IC_50_.

### Colony formation assay

GBC-SD, NOZ, and SGC-996 cells in logarithmic growth phase were enzymatically dissociated using 0.25% trypsin (Gibco) to obtain single-cell suspensions in culture medium. These single cells were then plated at a density of 400 cells per well in 6-well plates, with each group having 3 replicate wells. Subsequently, cells were exposed to varying concentrations of MGCD-265 for a duration of 10 days. Following this incubation period, the cell culture plates underwent several washes with PBS, fixation with 4% paraformaldehyde for 1 h, and staining with Rapid Giemsa staining kit (#E607314, Sangon Biotech (Shanghai) Co., Ltd.) for 30 min at room temperature. The resultant cell clones were quantified.

### Cell cycle analysis

Gallbladder cancer cells in logarithmic growth phase were seeded in six-well plates and treated with different concentrations of MGCD-265 for 24 h. Adherent cells were detached using trypsin digestion, yielding cell suspensions. These suspensions were then fixed in pre-chilled 70% ethanol overnight at 4 °C. Following centrifugation and removal of ethanol, cells were washed twice with cold PBS, resuspended, and treated with 100 µL RnaseA (1 mg/mL) and 400 µL propidium iodide (PI) staining buffer (KeyGEN BioTECH, #KGA9101-100) in accordance with the manufacturer’s instructions. After a 30-minute incubation at 4 °C in the dark, the cells were again washed twice with cold PBS, resuspended, and subjected to cell cycle analysis using flow cytometry (CytoFLEX LX, Beckman).

### Cell apoptosis analysis

Gallbladder cancer cells in logarithmic growth phase were cultured in 24-well plates (1.0 × 10^5^ cells/well) and exposed to varying concentrations of MGCD-265 for 24 h. Each concentration was tested in triplicate and a corresponding blank control was established. After a 24-hour incubation period, both adherent and supernatant cells were collected. A cell suspension was then prepared and mixed with 300 µL of Binding Buffer, 5 µL of Annexin V, and 5 µL of PI (KeyGEN BioTECH, # KGA1102-100). This suspension was incubated for 20 min at room temperature, protected from light. Apoptosis was measured using flow cytometry (CytoFLEX LX, Beckman).

### Statistical analysis

All data were presented as mean ± standard deviation (SD), with each experiment being replicated three times. Statistical analyses were performed using SPSS 22.0. An independent Student’s t-test was utilized for comparing two groups, while one-way analysis of variance (ANOVA) was employed for evaluating differences among multiple groups. A significance level of *p* < 0.05 was considered statistically significant.

## Results

### Analysis of differentially expressed genes and functions in gallbladder cancer through bioinformatics

**Fig. 1:**
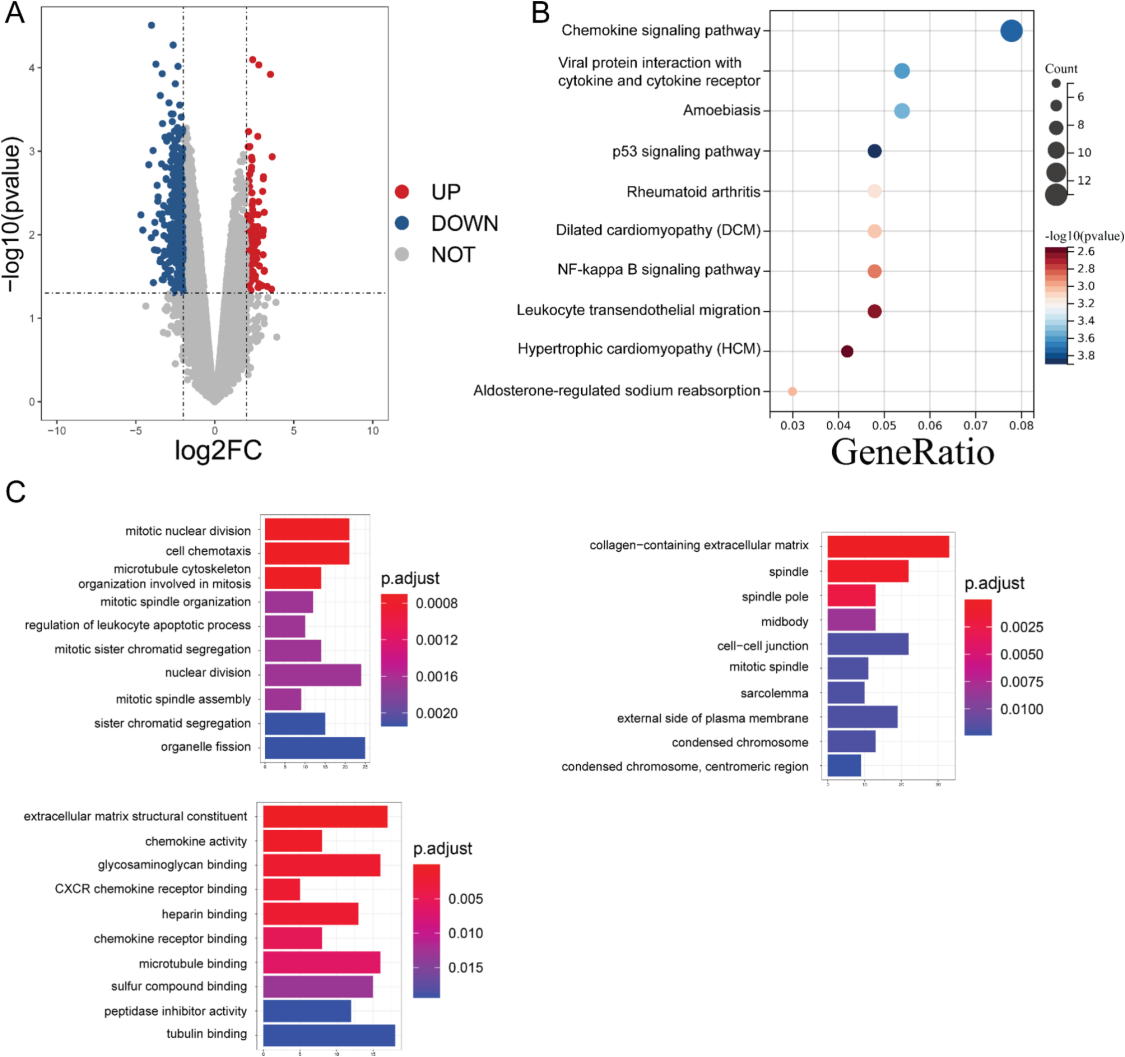
Bioinformatics analysis identifying genes and functions with significant differences in gallbladder cancer. (**A**) Gene expression analysis of cancer-adjacent tissues and cancer tissues in gallbladder cancer using the GEO database. (**B**) KEGG analysis of differentially expressed genes in gallbladder cancer. (**C**) GO analysis of differentially expressed genes in gallbladder cancer

To explore potential therapeutic interventions for gallbladder cancer, transcriptomic data from both gallbladder cancer tissues and adjacent tissues were extracted from online databases. Bioinformatics analysis was used to identify genes and functions that displayed differential expression in gallbladder cancer. Through a thorough investigation utilizing the GEO database, a total of 3553 genes were found to be significantly altered in gallbladder cancer tissues, with 1058 genes exhibiting significant upregulation and 2495 genes demonstrating significant downregulation (Fig. 1A). Subsequently, KEGG and GO analyses were employed to assess the differential genes, focusing on the top 10 enriched signaling pathway based on genes abundance. The KEGG analysis highlighted significant enrichment of differential genes in pathways such as the Chemokine signaling pathway, Viral protein interaction with cytokine and cytokine receptor, and Amoebiasis (Fig. 1B). GO functional annotation analysis revealed that the differential expressed genes exhibited significant enrichment in various pathways within the BP category, including mitotic nuclear division and cell chemotaxis. Similarly, within the MF category, the differential genes were predominantly enriched in pathways such as extracellular matrix structural constituent and chemokine activity. Furthermore, in the CC category, the differential genes demonstrated significant enrichment in pathways such as collagen-containing extracellular matrix and spindle (Fig. 1C).

Further analysis using PPI networks demonstrated a significant enrichment of the aforementioned differential genes (Fig. S1). Subsequent analyses were conducted on up-regulated and down-regulated genes, respectively. The results showed a prominent enrichment of up-regulated genes in the Cell cycle pathway, whereas down-regulated genes were mainly enriched in the Neuroactive ligand-receptor interaction pathway (Fig. S2).

### MST1R exhibits significant up-regulation in gallbladder cancer tissues, and its inhibitor, MGCD-265, demonstrates notable anti-gallbladder cancer effects

**Fig. 2:**
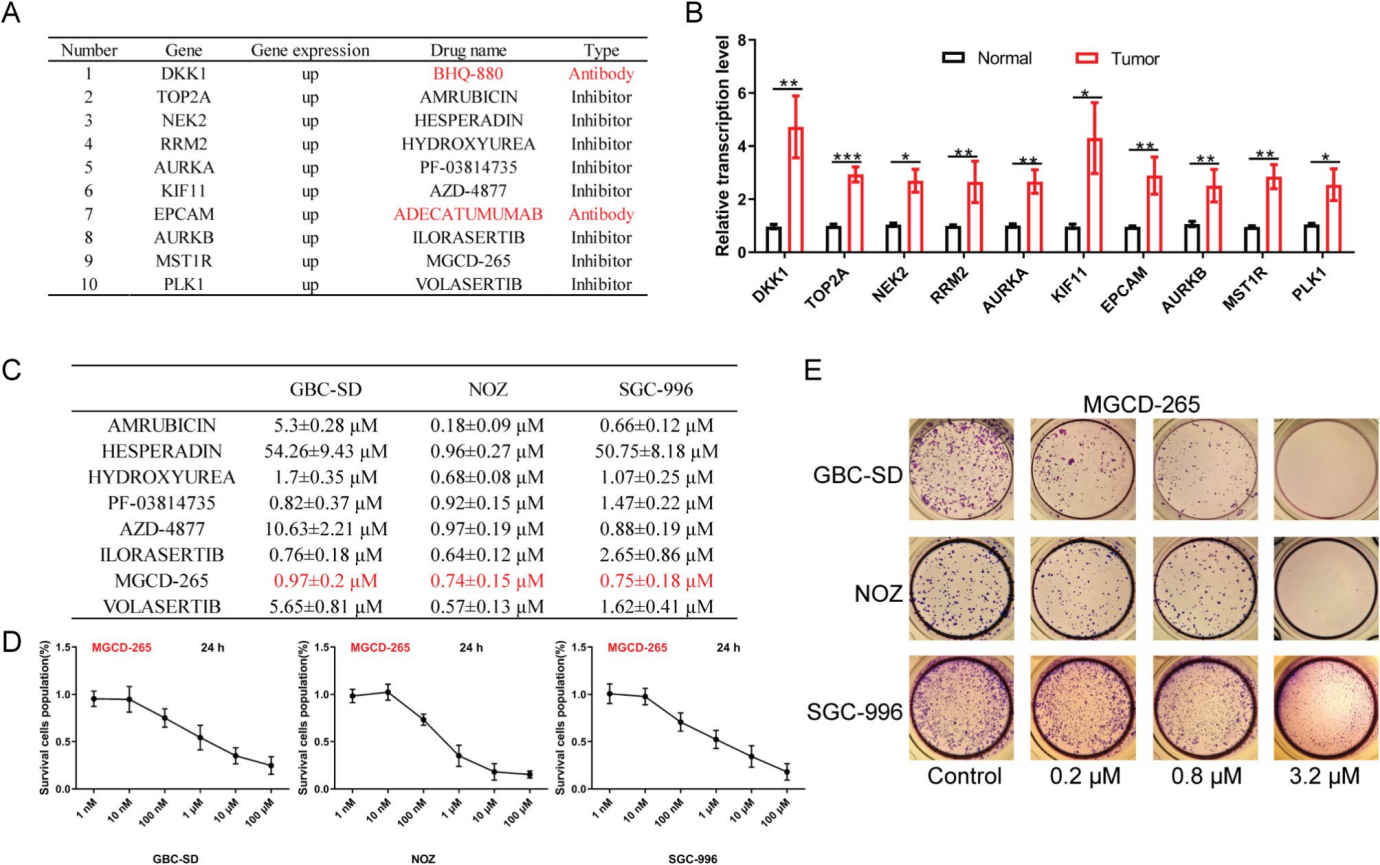
Significant upregulation of MST1R in gallbladder cancer and its inhibitor MGCD-265 exhibiting anti-tumor effects. (**A**) Bioinformatics analysis of relative transcription levels of genes. (**B**) Genes significantly upregulated in gallbladder cancer with inhibitors. (**C**) IC_50_ of the inhibitor on GBC-SD, NOZ, SGC-996 gallbladder cancer cells. (**D**) CCK-8 assay assessing the effect of MGCD-265 on the proliferative capacity of the three types of gallbladder cancer cells. (**E**) Colony formation assay evaluating the effect of MGCD-265 at 0.2 µM, 0.8 µM, and 3.2 µM on the colony formation capacity of the three types of gallbladder cancer cells. The cancer cells were subjected to varying doses of MGCD-265 over a 10-day period. *p < 0.05, **p < 0.01, ***p < 0.001

Through pharmacogenomic analysis targeting the up-regulated genes, we identified a specific set of inhibitors targeting DKK1, TOP2A, NEK2, RRM2, AURKA, KIF11, EPCAM, AURKB, MST1R, and PLK1 (Fig. 2A and B). To assess the antitumor effects of these inhibitors, we conducted CCK-8 assays. The results revealed varying degrees of inhibitory effects on the proliferation of GBC-SD, NOZ, and SGC-996 cells. PF-03814735 and ILORASERTIB exhibited IC_50_ values on GBC-SD of less than 1 µM, along with AMRUBICIN on NOZ and SGC-996 (Fig. S3). Moreover, AMRUBICIN showcased IC50 values against NOZ lower than 1 µM (Fig. S4), and AZD-4877 demonstrated IC50 values against SGC-996 also lower than 1 µM (Fig. S5). Notably, MGCD-265 exhibited IC50 values of 0.97 ± 0.2 µM, 0.74 ± 0.15 µM, and 0.75 ± 0.18 µM on GBC-SD, NOZ, and SGC-996 cells, respectively.

Given that the IC_50_ values of MGCD-265 were consistently below 1 µM for all three types of gallbladder cancer cells, it strongly suggests their heightened sensitivity to the MST1R inhibitor MGCD-265 (Fig. 2C). This relationship is visually depicted through the quantitative curves illustrating the action of MGCD-265 on the three types of gallbladder cancer cells (Fig. 2D). To further validate the biological activity of MGCD-265, we employed colony formation assay, confirming a dose-dependent inhibition of colony formation ability across the three gallbladder cancer cell lines (Fig. 2E). Furthermore, our findings reveal that the knockdown of MST1R significantly impeded the proliferation of GBC-SD cells and induced cell apoptosis, whereas the overexpression of MST1R promoted GBC-SD cell proliferation, without any observable alterations in cell apoptosis (Fig. S6).

### MGCD-265 induces G0/G1 phase arrest in gallbladder cancer cells

**Fig. 3:**
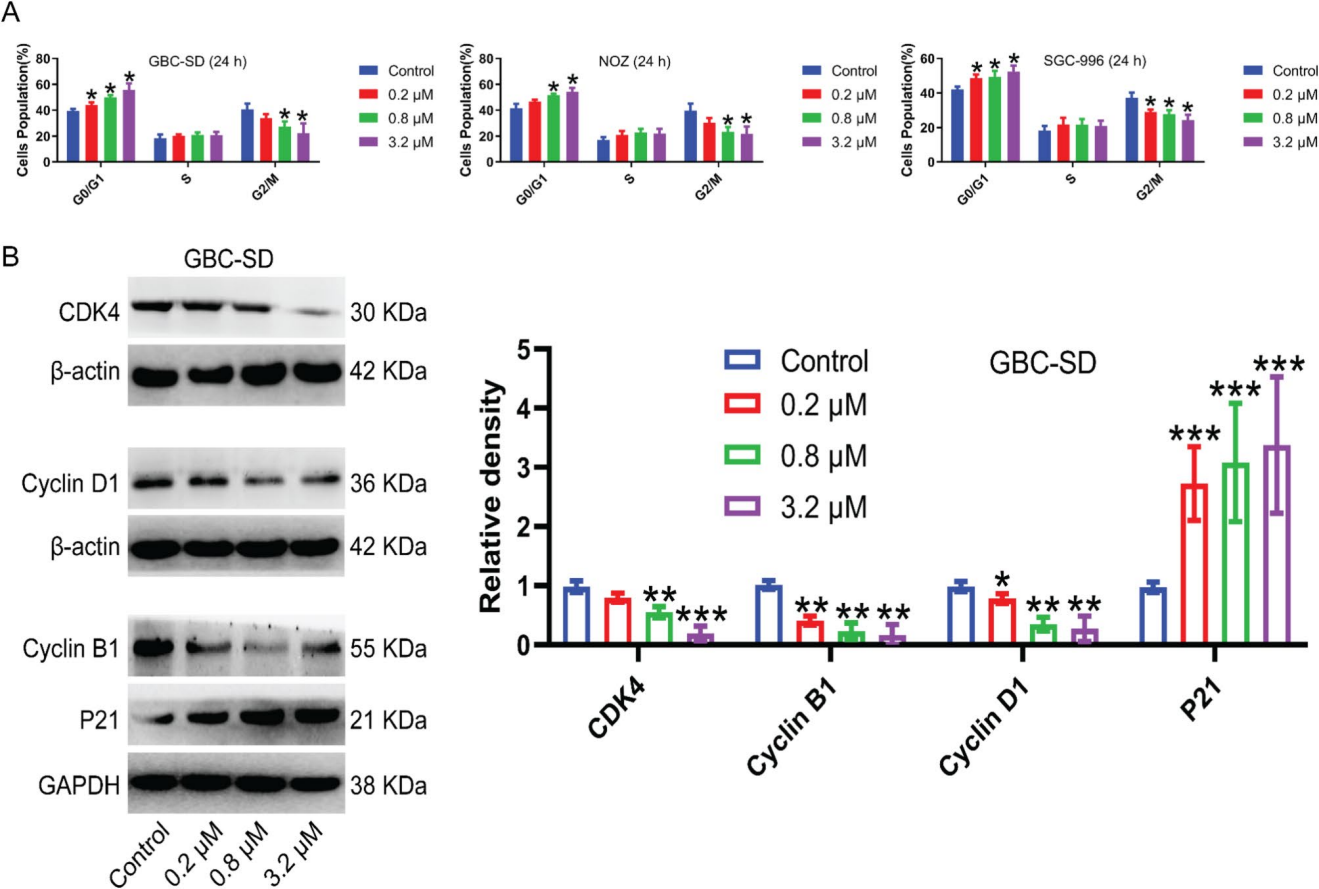
MGCD-265 induces G0/G1 phase arrest and alters the expression of cell cycle-related genes in gallbladder cancer cells. (**A**) Quantification of flow cytometric analysis. Gallbladder cancer cells (GBC-SD, NOZ, SGC-996) were treated with MGCD-265 for 24 h at concentrations of 0.2 µM, 0.8 µM, and 3.2 µM, respectively. (**B**) Western blot analysis of cell cycle-related proteins in GBC-SD cells after treatment with different concentrations of MGCD-265 for 24 h. *p < 0.05, **p < 0.01, ***p < 0.001

Considering its notable impact on inhibiting cell proliferation, a hypothesis was formulated linking this effect to the regulation of the cell cycle and apoptosis. To explore the mechanism by which MGCD-265 inhibits proliferation in GBC-SD, NOZ, and SGC-996 cells, flow cytometry was employed to evaluate cell cycle distribution. The findings demonstrated a significant increase in the distribution of cells in the G0/G1 phase in GBC-SD, NOZ, and SGC-996 cells after treatment with MGCD-265, accompanied by a dose-dependent decrease in the distribution of cells in the G2/M phase. Notably, there was no significant alteration in the S phase (Fig. 3A; Fig. S7). Moreover, examination of cell cycle-related proteins in GBC-SD cells through Western blotting demonstrated a significant downregulation in CDK4, Cyclin D1, and Cyclin B1 with escalating concentrations of MGCD-265. Conversely, the cell cycle inhibitor P21 exhibited a notable increase with the rising concentrations of MGCD-265 (Fig. 3B).

### MGCD-265 contributes to apoptosis in gallbladder cancer cells

**Fig. 4:**
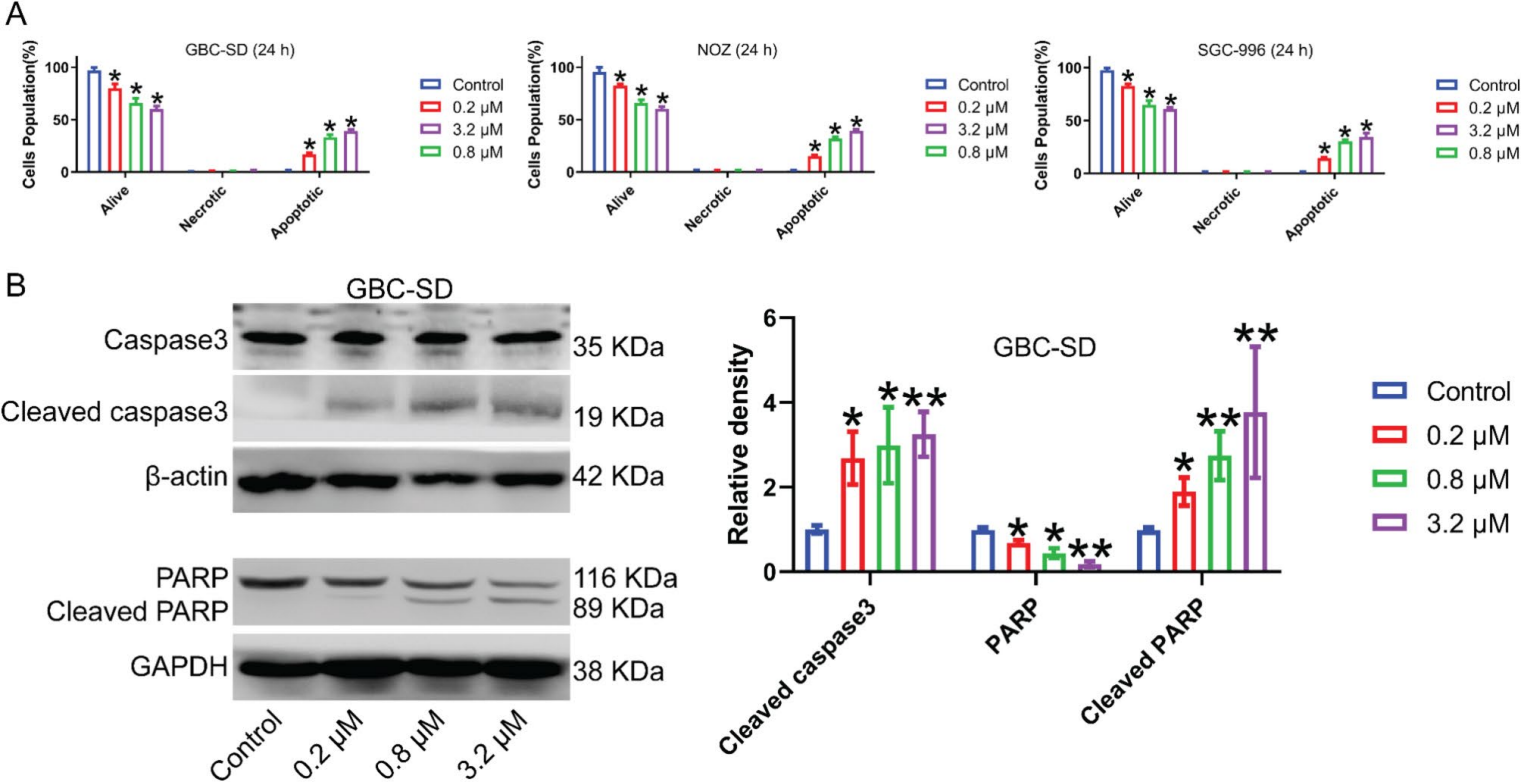
MGCD-265 causes apoptosis and regulates the expression of apoptosis-associated genes in gallbladder cancer cells. (**A**) Statistical analysis of cell apoptosis. Gallbladder cancer cells (GBC-SD, NOZ, SGC-996) were treated with MGCD-265 for 24 h at concentrations of 0.2 µM, 0.8 µM, and 3.2 µM, respectively. (**B**) Western blot analysis of expression levels of apoptosis-related proteins in GBC-SD cells after treatment with different concentrations of MGCD-265 for 24 h. *p < 0.05, **p < 0.01

We employed flow cytometry to evaluate the effect of MGCD-265 on apoptosis in GBC-SD, NOZ, and SGC-996 cells. The results indicated a notable decrease in viable cell counts across all three gallbladder cancer cell types as the concentration of the drug increased following MGCD-265 treatment. Additionally, there was a substantial increase in the population of apoptotic cells that correlated with the elevation in drug concentration (Fig. 4A; Fig. S8). These findings suggest that MGCD-265 induces apoptosis in GBC cells in a dose-dependent manner. Next, we assessed the expression of apoptosis-related protein in GBC-SD cells using Western blotting. Notably, the level of PARP exhibited a marked decrease with elevated MGCD-265 concentration, whereas Cleaved PARP exhibited a significant increase in concentration in response to higher MGCD-265 doses. Additionally, the concentration of Cleaved caspase-3, a crucial executor of apoptosis, demonstrated a substantial increase proportionate to MGCD-265 concentration (Fig. 4B).

### MGCD-265 exhibits *in vivo *antitumor effects against gallbladder cancer

**Fig. 5:**
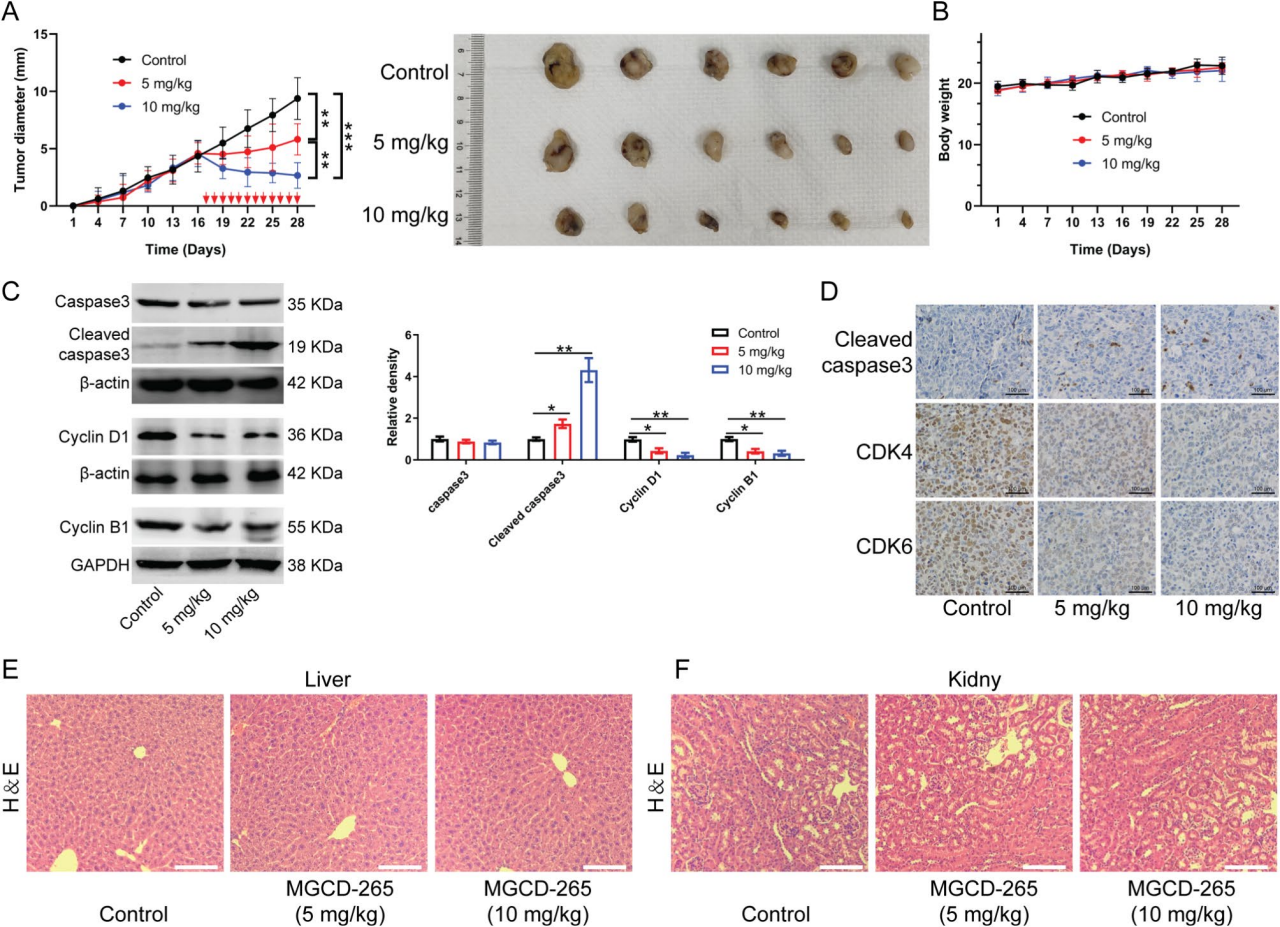
Subcutaneous xenografts in nude mice confirm the in vivo anti-gallbladder cancer effect of MGCD-265. (**A**) Tumor growth after treatment with 5 mg/kg or 10 mg/kg MGCD-265. (**B**) Effect of different doses of MGCD-265 treatment on the body weight of tumor-bearing mice. (**C**) Western blotting showing cell cycle and apoptosis-related protein levels in tumor tissues of mice after treatment with different doses of MGCD-265. (**D**) Immunohistochemical staining of Cleaved caspase3, Cyclin D1, and Cyclin B1 in tumor nodules after treatment with different doses of MGCD-265. (E, F) HE staining evaluating the pathological status of the liver and kidney in tumor-bearing mice after treatment with different doses of MGCD-265. *p < 0.05, **p < 0.01, ***p < 0.001

We established a Cell line-derived xenograft (CDX) mouse model using GBC-SD cells, initiating drug intervention once the tumor diameter reached approximately 5 mm. Both 5 mg/kg and 10 mg/kg of MGCD-265 significantly decreased the diameter and volume of subcutaneous tumors in mice, showcasing a dose-dependent inhibition of gallbladder cancer cell growth in vivo (Fig. 5A). Importantly, the administration of MGCD-265 at these doses had no notable impact on the body weight of the mice (Fig. 5B).

Next, Western blotting revealed that Caspase3 exhibited a gradual decrease with increasing dose of MGCD-265, while Cleaved caspase3 levels exhibited a significant increase in correlation with MGCD-265 dose. Concurrently, cell cycle-related proteins Cyclin D1 and Cyclin B1 showed a substantial decrease as the dose of MGCD-265 increased (Fig. 5C). Subsequent immunohistochemical analysis of subcutaneous tumor tissues obtained from mice administered with 5 mg/kg or 10 mg/kg of MGCD-265 revealed a reduction in levels of CDK4 and CDK6, accompanied by an elevation in levels of Cleaved caspase3 (Fig. 5D). Additionally, we assessed the impact of MGCD-265 treatment on liver and kidney tissues of the mice. Hematoxylin and Eosin staining demonstrated that MGCD-265 treatment did not induce significant adverse effects on the liver and kidney tissues of the mice (Fig. 5E and F).

### Transcriptomics analysis elucidates the multifaceted intracellular pathways linked to MGCD-265 treatment

**Fig. 6:**
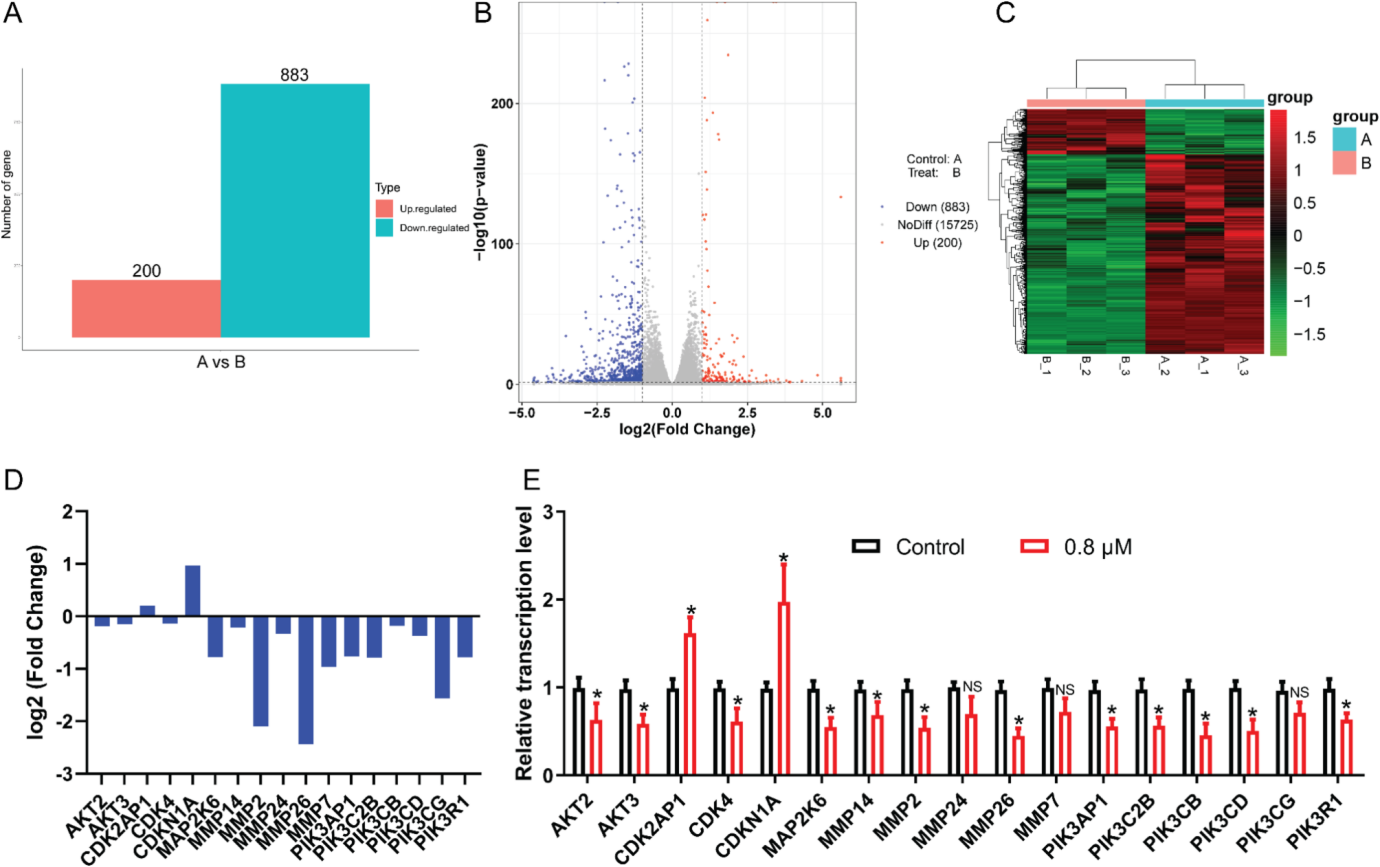
Transcriptomics reveals the association of anti-gallbladder cancer effect of MGCD-265 with multiple intracellular pathways. (**A**) Transcriptomic sequencing determines the number of differentially expressed genes in GBC-SD gallbladder cancer cells treated with 0.8 µM MGCD-265 for 24 h. (**B**) Volcano plot depicting differentially expressed genes in gallbladder cancer cells after 24-hour treatment with MGCD-265. (**C**) Heatmap depicting differentially expressed genes in gallbladder cancer cells after 24-hour treatment with MGCD-265. (**D**) Effect of 24-hour MGCD-265 treatment on the expression of differentially expressed genes. (**E**) RT-qPCR analysis assessing the effect of MGCD-265 treatment for 24 h on gene expression in gallbladder cancer cells. NS, not significant; *p < 0.05

The preceding experiments provided initial validation of MGCD-265, showing noticeable effects against gallbladder cancer. To further understand its mechanism against gallbladder cancer, we examined its impact on the transcriptome of gallbladder cancer cells. The analysis revealed 200 transcripts exhibiting a notable increase in abundance post MGCD-265 treatment, contrasting with 883 transcripts displaying a significant decrease (Fig. 6A and B). Heatmap clustering analysis underscored distinctive patterns between the MGCD-265-treated and untreated groups (Fig. 6C). Moreover, we tested the expression of several differentially expressed genes. Data unveiled a substantial upregulation of CDK2AP1 and CDKN1A expression, alongside a noteworthy downregulation of MMP2 and MMP26 following MGCD-265 treatment (Fig. 6D). The differentially expressed genes were further validated using the RT-qPCR, confirming the congruence of expression patterns of genes, including AKT2, AKT3, and CDK2AP1, with the transcriptomic sequencing results (Fig. 6E).

Insightful GO analysis indicated that these differentially expressed genes were predominantly enriched in molecular functions, such as GO:0071944 cell periphery, and were associated with cellular components like GO:0005201 extracellular matrix structural constituent, and GO:0007155 cell adhesion, encompassing various biological processes (Fig. S9A). Additionally, KEGG analysis unveiled a significant enrichment of the differentially expressed genes in pathways related to Protein digestion and absorption, Organismal Systems, and Environmental Information Processing (Fig. S9B). Further dissection demonstrated distinctive enrichment patterns in different pathways for down-regulated (Fig. S10) and up-regulated genes (Fig. S11).

### Synergistic anti-tumor effects of MST1R inhibitors in combination with JMJD6 inhibitors in mice

**Fig. 7:**
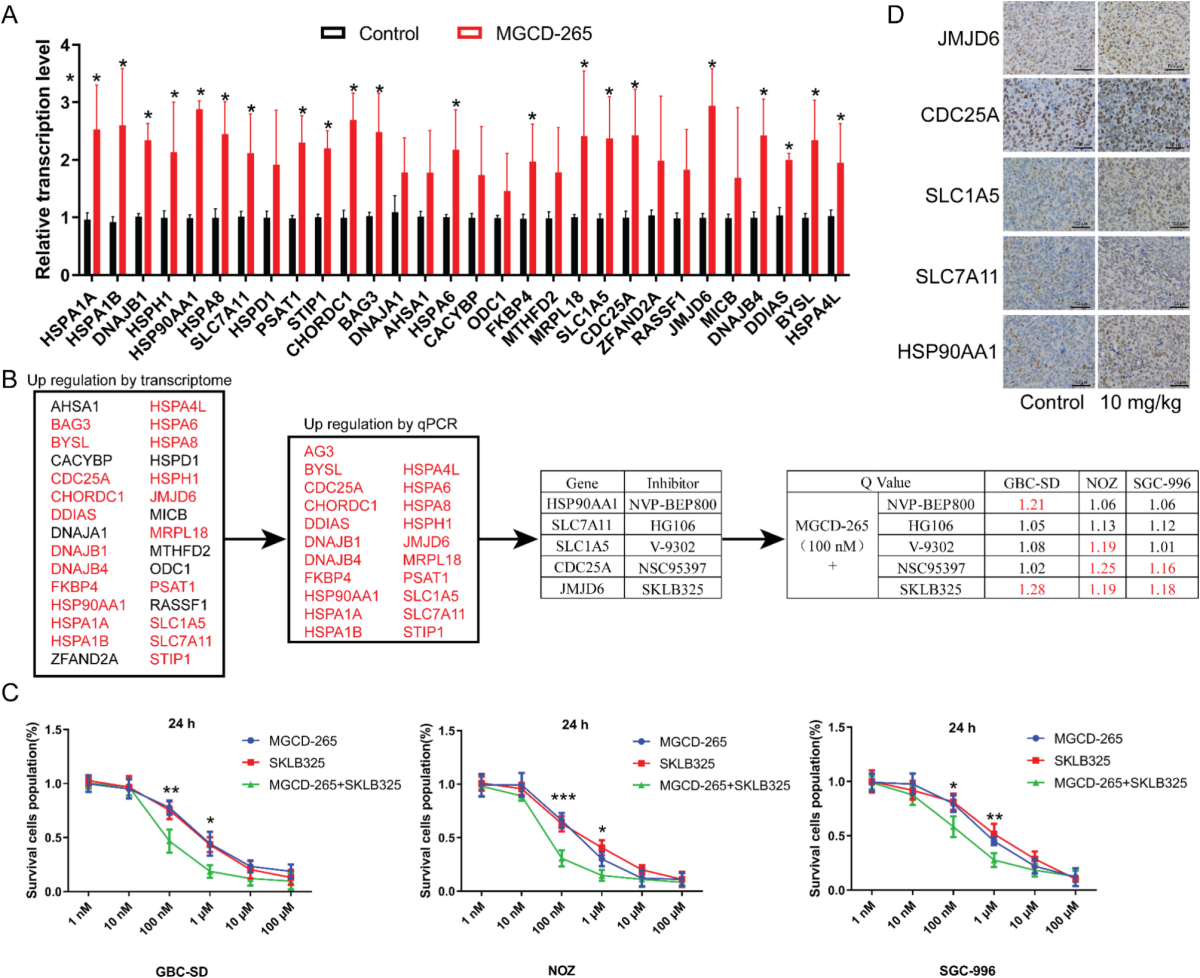
Synergistic antitumor effect of MST1R inhibitor and JMJD6 inhibitor combination in vitro. (**A**) RT-qPCR validation of transcription levels of the top 30 upregulated genes from transcriptomic sequencing. (**B**) Schematic representation of drug screening and statistical results of drug interaction Q values. (**C**) Effect of MGCD-265 in combination with SKLB325 on in vivo proliferative capacity of gallbladder cancer cells GBC-SD, NOZ, SGC-996. (**D**) Immunohistochemical staining illustrating the expression levels of JMJD6, CDC25A, HSP90AA1, SLC7A11, and SLC1A5 in subcutaneous tumor tissues of mice treated with 10 mg/kg MGCD-265. *p < 0.05, **p < 0.01, ***p < 0.001

To identify combination therapeutic targets in MST1R inhibitor MGCD-265-treated gallbladder cancer cells, we verified the top 30 up-regulated genes from transcriptomics sequencing using RT-qPCR. We found that the expression of 21 genes, including HSPA1A and HSPA1B, was significantly up-regulated, whereas 9 genes, including HSPD1, showed no significant changes (Fig. 7A). The analysis of these 21 significantly upregulated genes led to the identification of 5 relevant small molecule inhibitors. These five small molecule inhibitors were used in combination with MGCD-265 to treat gallbladder cancer cells, respectively. It was observed that the Q values of MGCD-265 in combination with SKLB325, a JMJD6 inhibitor, were all greater than 1.15 for all three types of gallbladder cancer cells (Fig. 7B and C; Fig. S12), suggesting that MGCD-265 in combination with SKLB325 might have a more potent anti-tumor effect. Immunohistochemical staining was further conducted on the subcutaneous tumor tissues of the aforementioned mice treated with 10 mg/kg MGCD-265, indicating an up-regulation of the protein expression levels of JMJD6, CDC25A, HSP90AA1, SLC7A11, and SLC1A5 compared to the control (Fig. 7D).

**Fig. 8:**
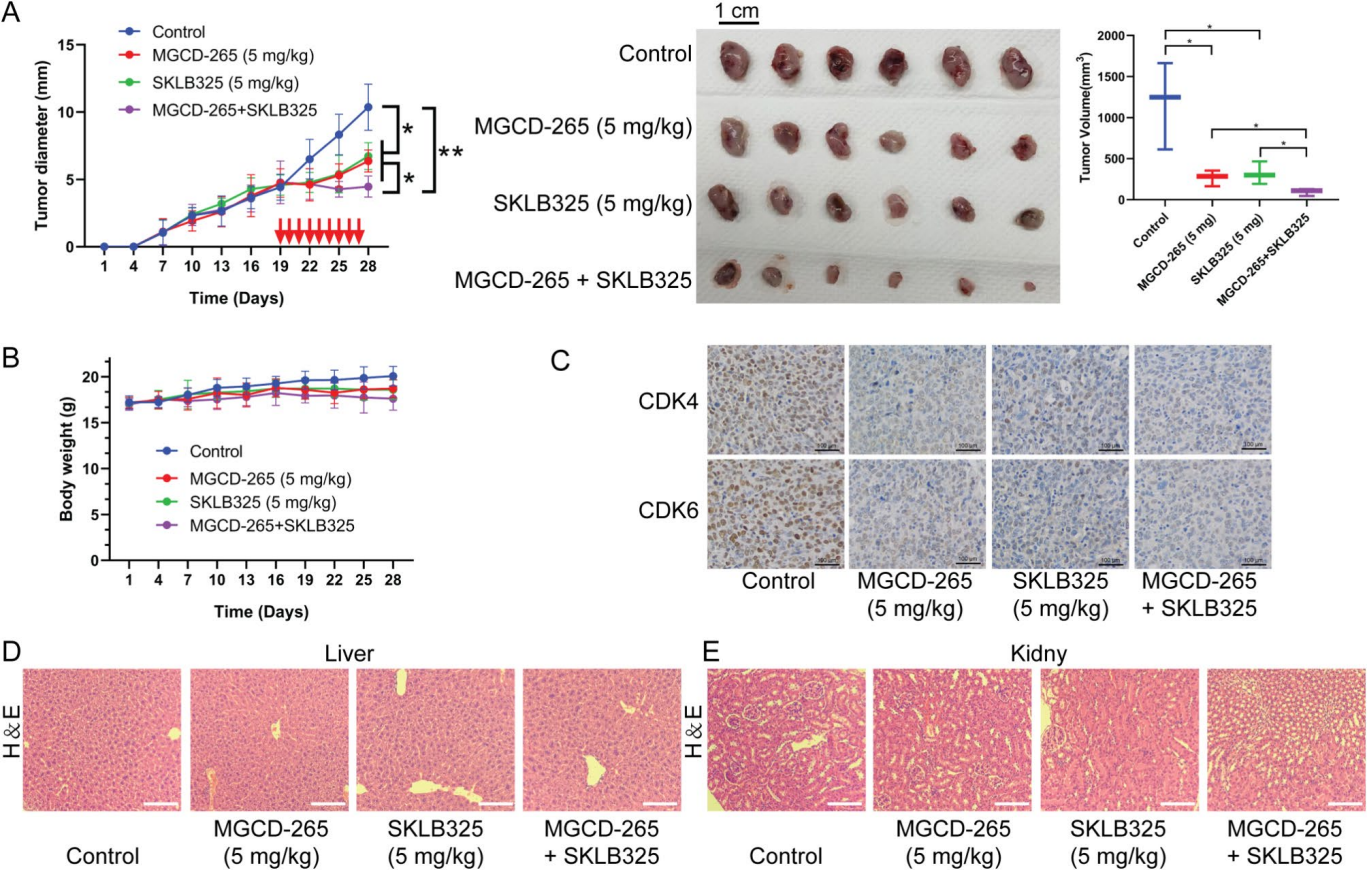
In vivo experimental validation of the synergistic anti-tumor effect of MST1R inhibitor in combination with JMJD6 inhibitor. (**A**) Subcutaneous tumor volume in nude mice treated with a combination of 5 mg/kg MGCD-265 and 5 mg/kg SKLB325. (**B**) Effect of combined treatment of MGCD-265 and SKLB325 on the body weight of tumor-bearing mice. (**C**) Immunohistochemical staining for Cyclin D1 and Cyclin B1 in tumor nodules of tumor-bearing mice after combined treatment with MGCD-265 and SKLB325. (D, E) HE staining to assess the effect of combined MGCD-265 and SKLB325 treatment on the hepatic and renal tissue morphology of tumor-bearing mice. *p < 0.05, **p < 0.01

In vitro experimental results confirmed the potential for a synergistic effect with the combined use of MST1R inhibitor and JMJD6 inhibitor. Therefore, this study further confirmed whether the combination of MGCD-265 and SKLB325 had a similar synergistic effect on tumor growth. The results demonstrated that the combination of MGCD-265 or SKLB325 significantly reduced the growth capacity of gallbladder cancer cells compared to treatment with MGCD-265 or SKLB325 alone (Fig. 8A). Meanwhile, treatment with MGCD-265 or SKLB325 alone had no significant effect on the body weight of the mice. Although MGCD-265 in combination with SKLB325 reduced the body weight of mice, there was no significant difference (Fig. 8B). Immunohistochemical staining revealed that MGCD-265 combined with SKLB325 significantly reduced the levels of CDK4 and CDK6 compared to treatment with MGCD-265 or SKLB325 alone (Fig. 8C), and HE staining showed no significant alteration in the morphology of the liver and kidney tissues (Fig. 8D and E).

To elucidate the underlying mechanisms by which MST1R regulates JMJD6, GBC-SD cells were treated with MST1R inhibitors for 48 h. The results indicated significant alterations in the protein levels of β-catenin [15] and associated molecules, specifically an increase in β-catenin phosphorylation and a decrease in its overall abundance. Conversely, the expression levels of NF-κB-related proteins remained largely unchanged, with no significant differences observed in the levels of IκBs, IκBs phosphorylation, P65 expression, P65 phosphorylation, P50 expression, or P50 phosphorylation (Fig. S13). These findings indicate that MST1R may influence downstream gene expression through the regulation of β-catenin signaling pathways.

## Discussion

In gallbladder cancer and several other tumor tissues, the expression of MET, a tyrosine kinase, is markedly up-regulated [16, 17]. Inhibitors targeting MET have demonstrated significant effectiveness in inhibiting proliferation, migration, and invasion of gallbladder cancer cells [18, 19]. MST1R, a homologue of MET, orchestrates cell signaling pathways that foster tumorigenesis, cancer cell survival, and growth [2, 20, 21]. MST1R is notably overexpressed in breast cancer, colorectal cancer, pancreatic cancer, and prostate cancer, exerting a tumor-promoting effect through diverse mechanisms [22–25]. It is consistently observed that heightened expression and/or activation of MST1R is associated with unfavorable patient prognosis [21]. Multiple isoforms of MST1R have been identified, exhibiting variations in structure, activation, and pathway regulation. Preclinical studies and clinical trials have validated the efficacy of small molecule inhibitors targeting both MST1R and MET [26–28]. In this study, we screened MGCD-265, an MST1R inhibitor exhibiting anti-gallbladder cancer effects. MGCD-265 demonstrates anti-tumor effects in vitro through apoptosis induction and cell cycle arrest. Moreover, it consistently inhibits gallbladder cancer cell growth in vivo in a dose-dependent manner, underscoring the potential of MGCD-265, an MST1R inhibitor, to evolve into a potent anti-gallbladder cancer drug.

The PI3K/AKT/mTOR pathway undergoes significant upregulation in various cancers, including gallbladder cancer [29] and breast cancer [30], playing a pivotal role in promoting tumor progression. MET, upon binding to hepatocyte growth factor (HGF), activates the downstream PI3K/AKT signaling pathway, thereby influencing critical biological processes such as cell proliferation, migration, and drug resistance [31], corroborating findings in our study. Mechanistically, MST1R inhibitors predominantly exert anti-tumor effects by suppressing the expression of downstream genes, including members of the MMP family or genes associated with the PI3K pathway. However, notably, this inhibition leads to a notable upregulation in the expression of pro-carcinogenic genes such as HSP90AA1, DNAJB1, and JMJD6. This phenomenon might be attributed to a compensatory pathway initiated by tumor cells post stress signal exposure, aimed at evading cell dysfunction and enhancing their survival. We speculate that this could potentially serve as a mechanism for the emergence of drug resistance in response to small molecule drugs during tumor treatment, thereby presenting a plausible target for the development of combination therapeutic strategies in the treatment of gallbladder cancer.

Epigenetic modifications play a pivotal role in tumorigenesis, with histone demethylase inhibitors demonstrating noteworthy anticancer properties across a spectrum of tumor types [32–34]. Notably, previous works have reported a significant upregulation of JMJD6 in breast cancer tissues, and specific small molecule inhibitors targeting JMJD6 have exhibited a reduction in the proliferative capacity of breast cancer cells both in vitro and in vivo [35–37]. In this study, we corroborated a substantial upregulation of JMJD6 expression subsequent to MST1R inhibitor treatment. Furthermore, the concomitant utilization of MST1R and JMJD6 inhibitors manifested a synergistic anti-gallbladder cancer effect. Importantly, safety analyses indicated no significant adverse effects on liver and kidney tissues of the mice subjects with either the MST1R inhibitor, the JMJD6 inhibitor alone, or their combination, affirming a high safety profile. However, it is imperative to acknowledge certain limitations within this study, notably the potential off-target effects of MST1R inhibitors, concurrently inhibiting other target proteins. Additionally, the comprehensive impact of drug combinations on gene expression and signaling pathways in tumor cells remains incompletely explored. Despite these constraints, our present findings underscore a highly promising avenue for gallbladder cancer treatment. Future research endeavors will delve into elucidating the molecular mechanisms underpinning the combinatorial drug approach, employing diverse methodologies to enhance the anti-gallbladder cancer efficacy. These forthcoming insights are anticipated to fortify the theoretical basis for clinical application of these drugs.

## Conclusions

In summary, this study explored the specific mechanism of MGCD-265 and the feasibility of combination therapy, confirming that the MST1R inhibitor MGCD-265 holds promise as a potential anti-gallbladder cancer drug. The combined use of MST1R and JMJD6 inhibitors significantly enhances the anti-gallbladder cancer effect, potentially offering a viable combination therapy strategy for gallbladder cancer treatment.

### Electronic supplementary material

Below is the link to the electronic supplementary material.

Supplementary Material 1

Supplementary Material 2

Supplementary Material 3

Supplementary Material 4

Supplementary Material 5

Supplementary Material 6

Supplementary Material 7

Supplementary Material 8

Supplementary Material 9

Supplementary Material 10

Supplementary Material 11

Supplementary Material 12

Supplementary Material 13

Supplementary Material 14

## Data Availability

The datasets used and/or analysed during the current study are available from the corresponding author on reasonable request.

## Abbreviations

GBC: Gallbladder Cancer
GO: Gene Ontology
HGF: Hepatocyte Growth Factor
KEGG: Kyoto Encyclopedia of Genes and Genomes
TCGA: The Cancer Genome Atlas
