# Supplementary figures and images for: MST1R-targeted therapy in the battle against gallbladder cancer
Source: Cell Biosci. 2024 Aug 29;14:109. doi: 10.1186/s13578-024-01290-w (PMC11363441; doi:10.1186/s13578-024-01290-w)

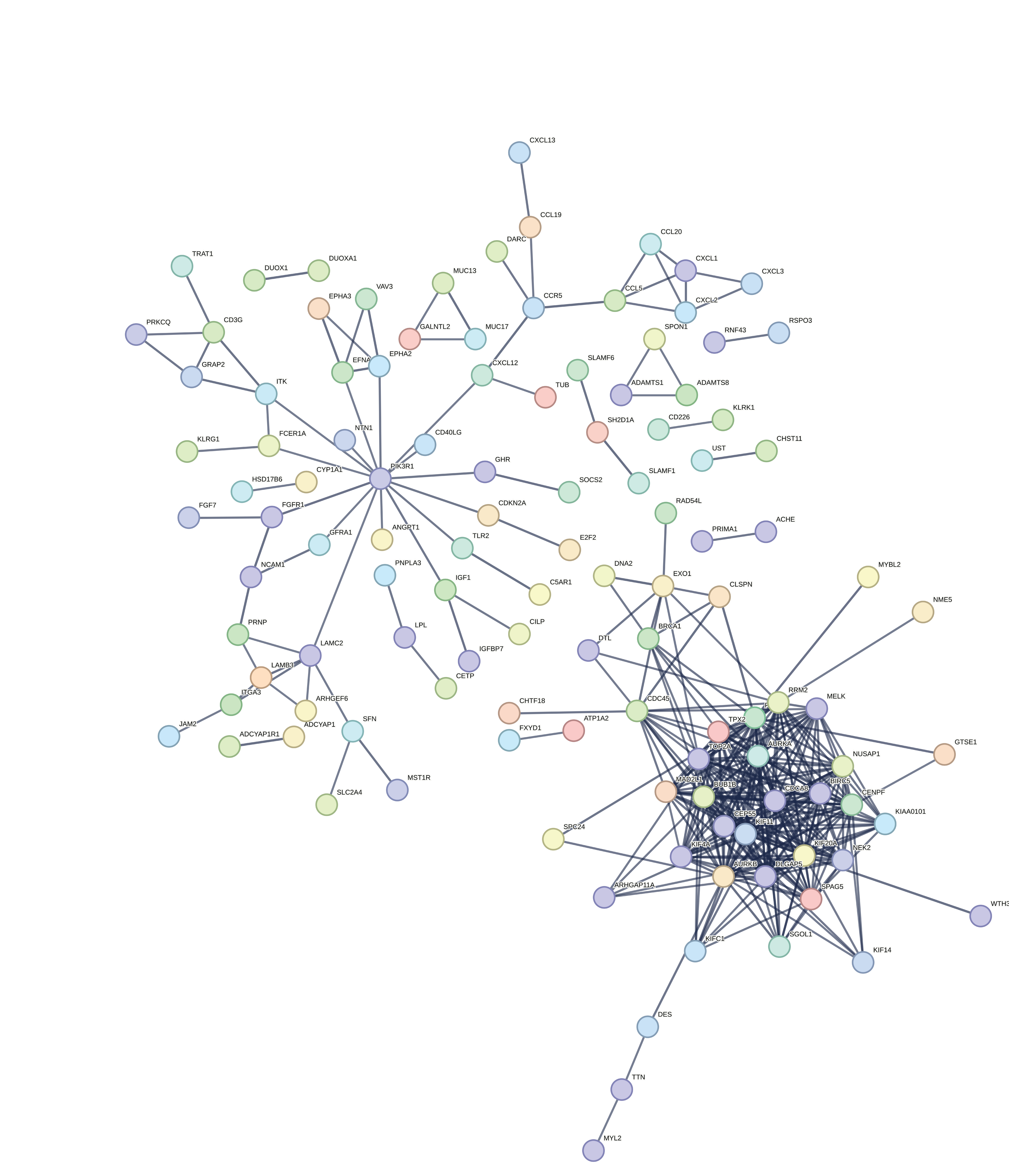

Supplement: Supplementary file 1 — Supplementary Material 1 [file 13578_2024_1290_MOESM1_ESM.tif]

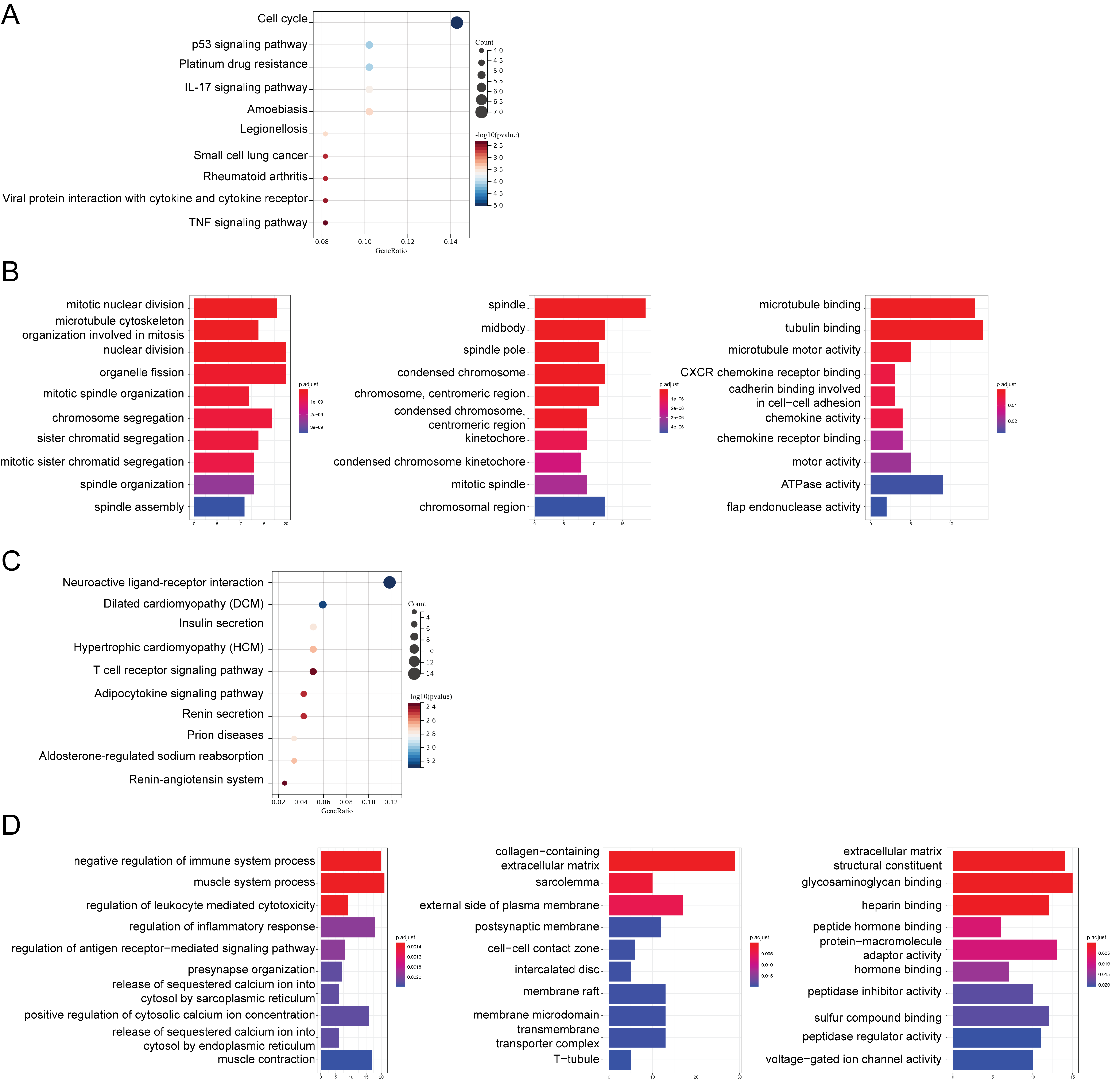

Supplement: Supplementary file 2 — Supplementary Material 2 [file 13578_2024_1290_MOESM2_ESM.tif]

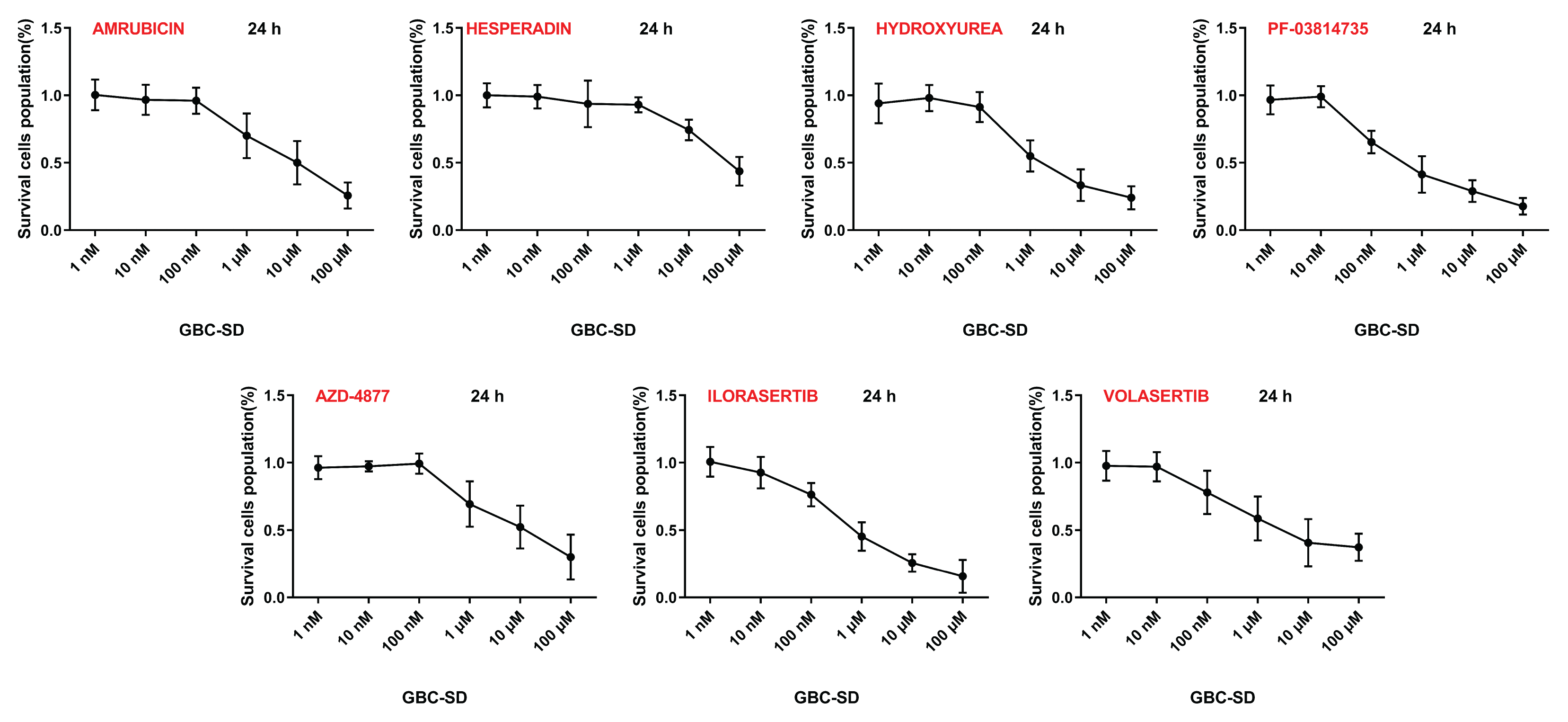

Supplement: Supplementary file 3 — Supplementary Material 3 [file 13578_2024_1290_MOESM3_ESM.tif]

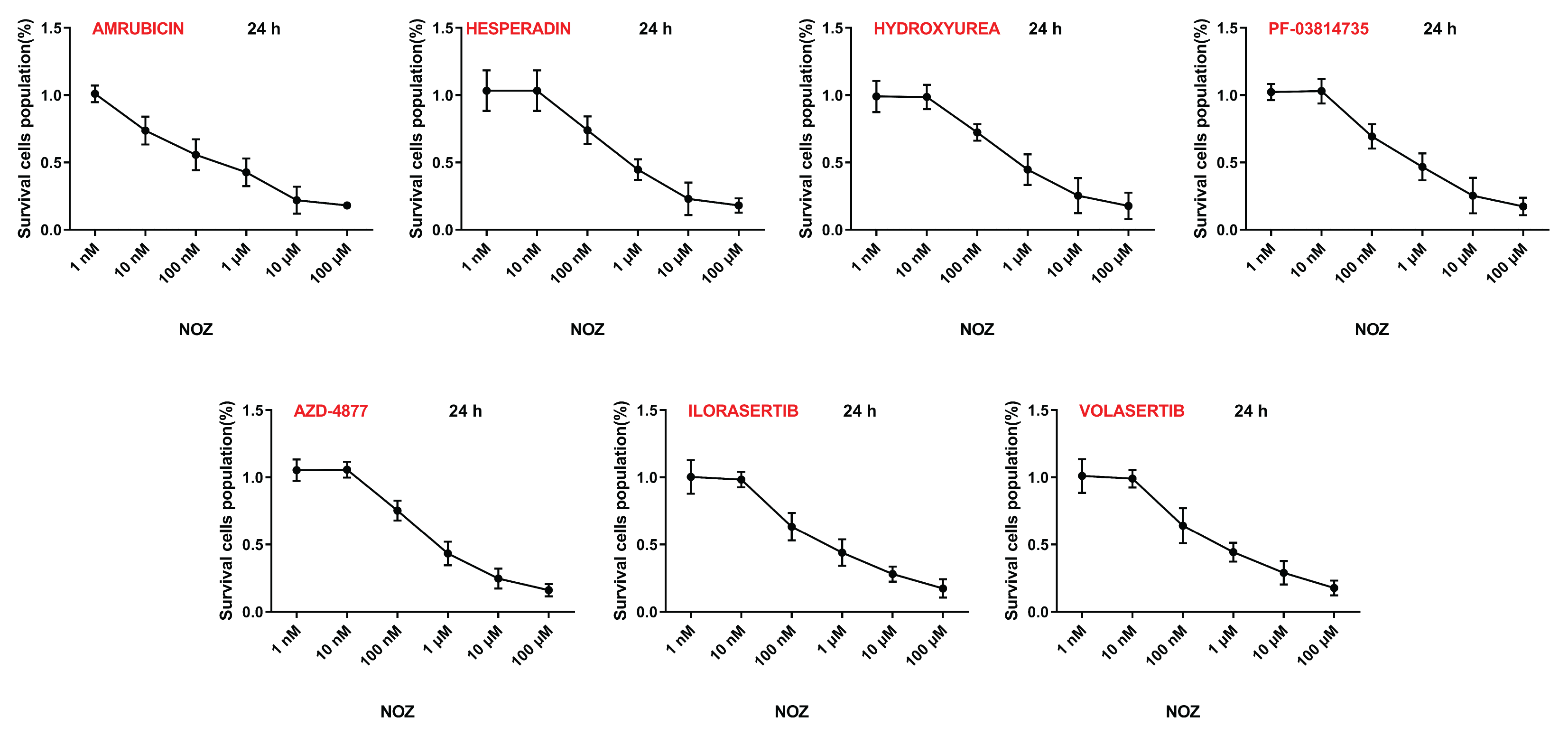

Supplement: Supplementary file 4 — Supplementary Material 4 [file 13578_2024_1290_MOESM4_ESM.tif]

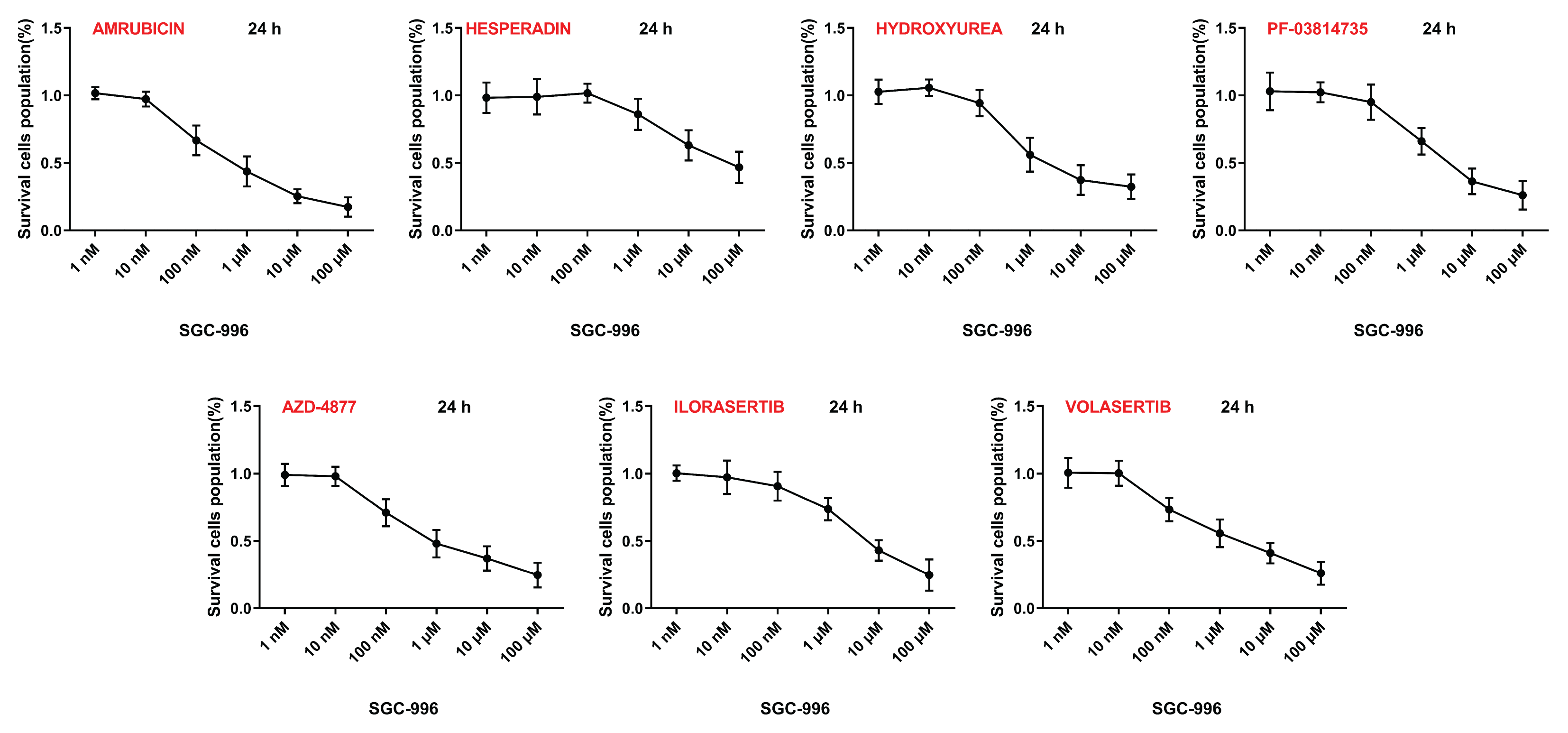

Supplement: Supplementary file 5 — Supplementary Material 5 [file 13578_2024_1290_MOESM5_ESM.tif]

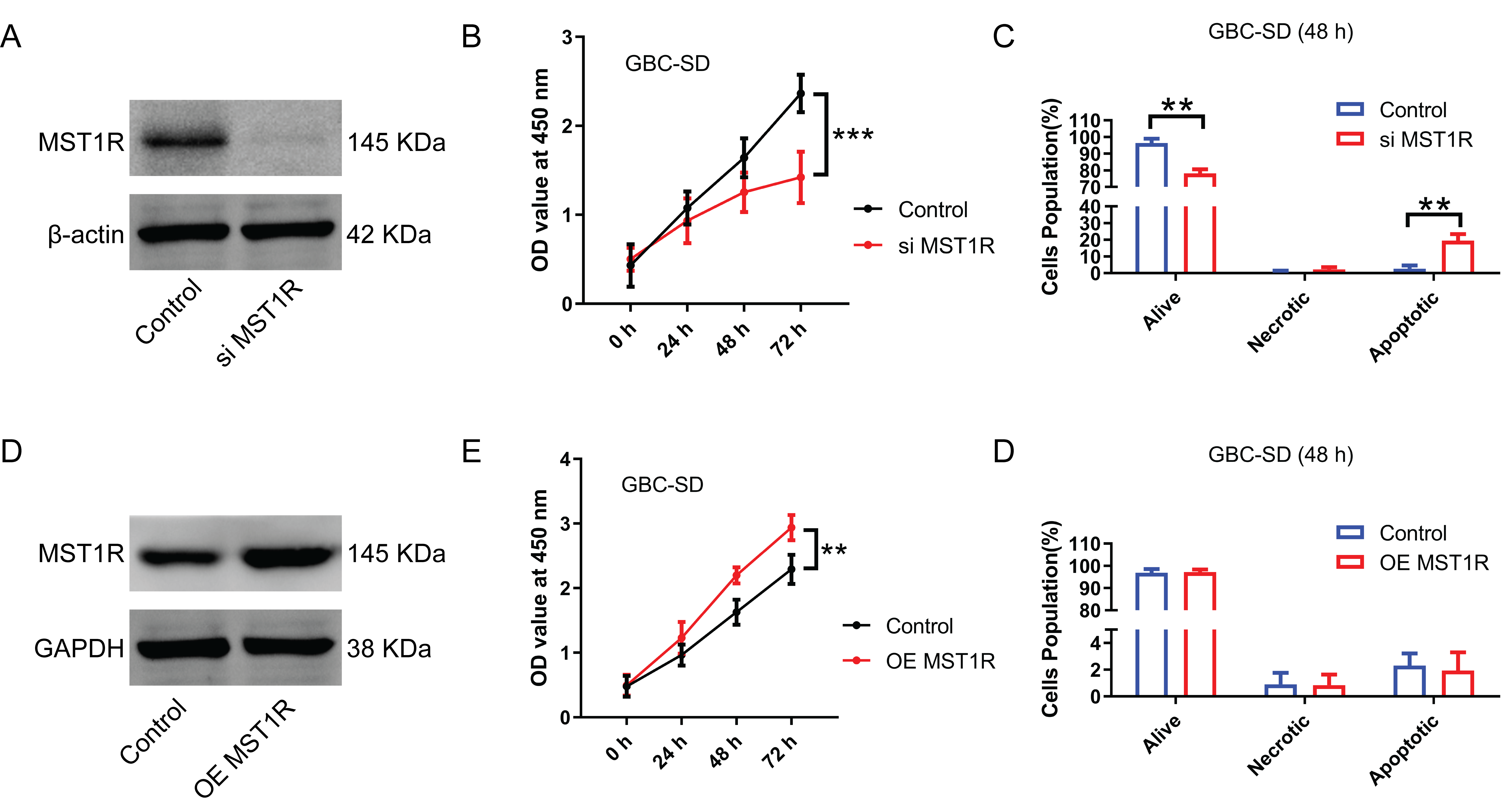

Supplement: Supplementary file 6 — Supplementary Material 6 [file 13578_2024_1290_MOESM6_ESM.tif]

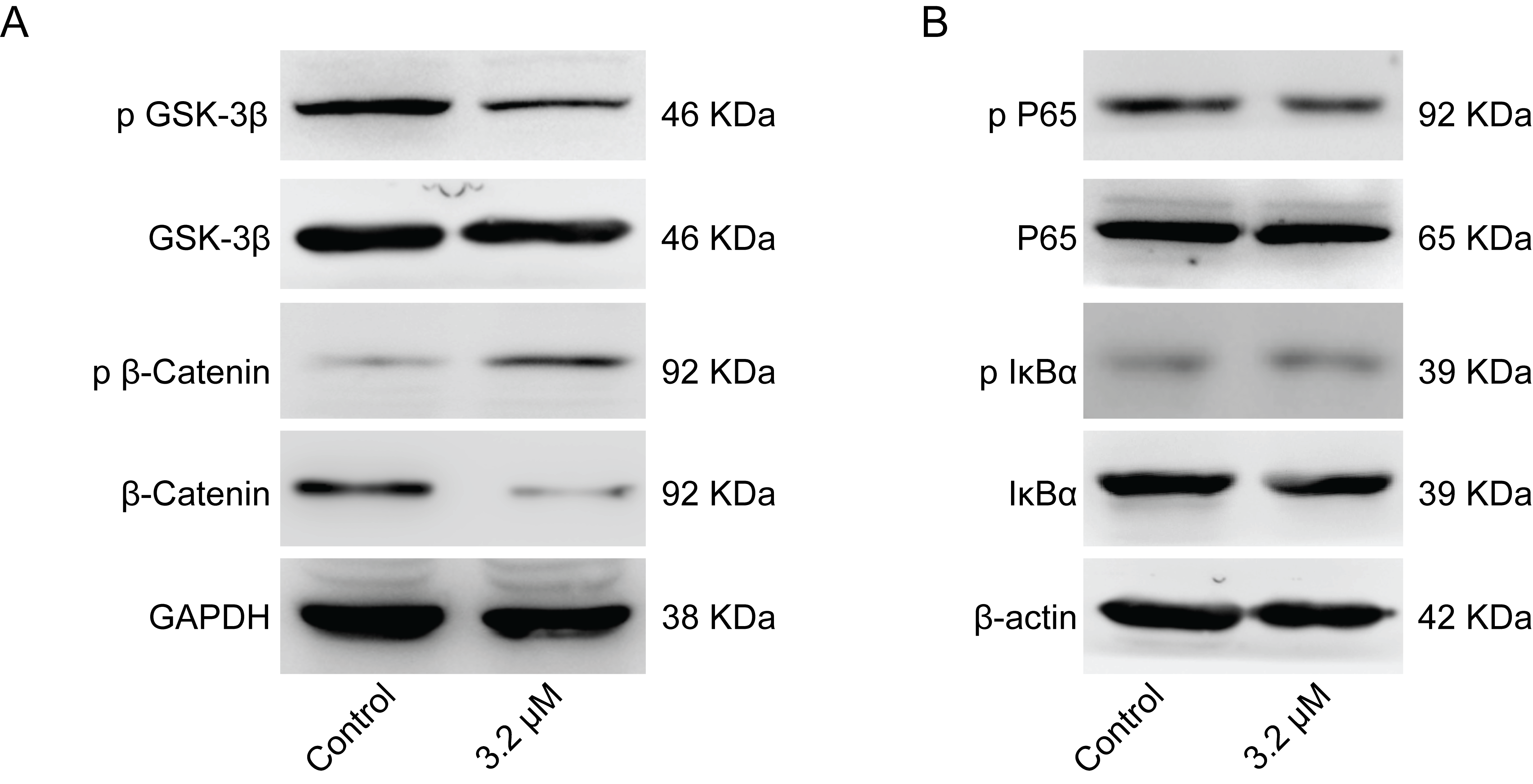

Supplement: Supplementary file 7 — Supplementary Material 7 [file 13578_2024_1290_MOESM7_ESM.tif]

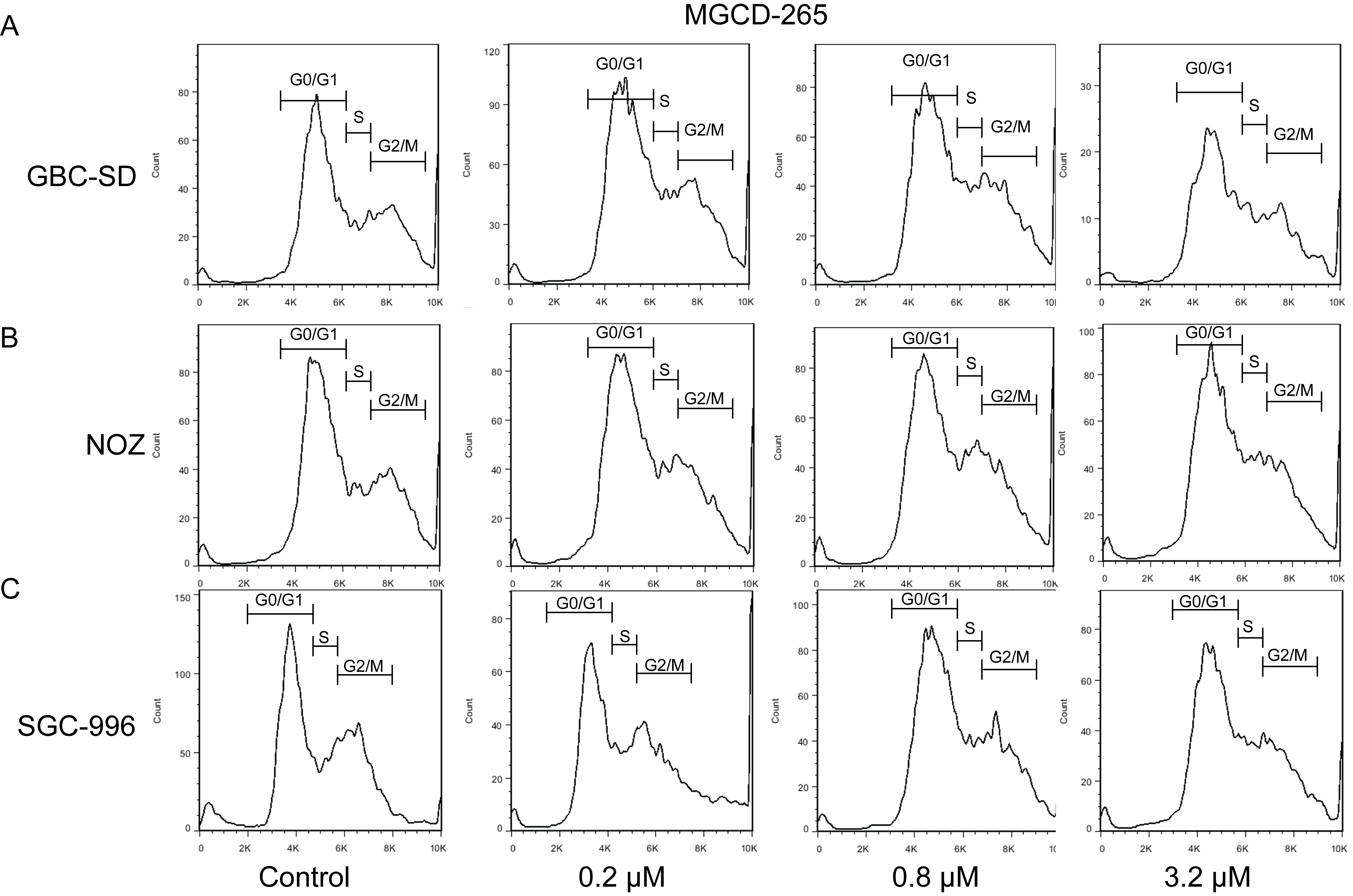

Supplement: Supplementary file 8 — Supplementary Material 8 [file 13578_2024_1290_MOESM8_ESM.tif]

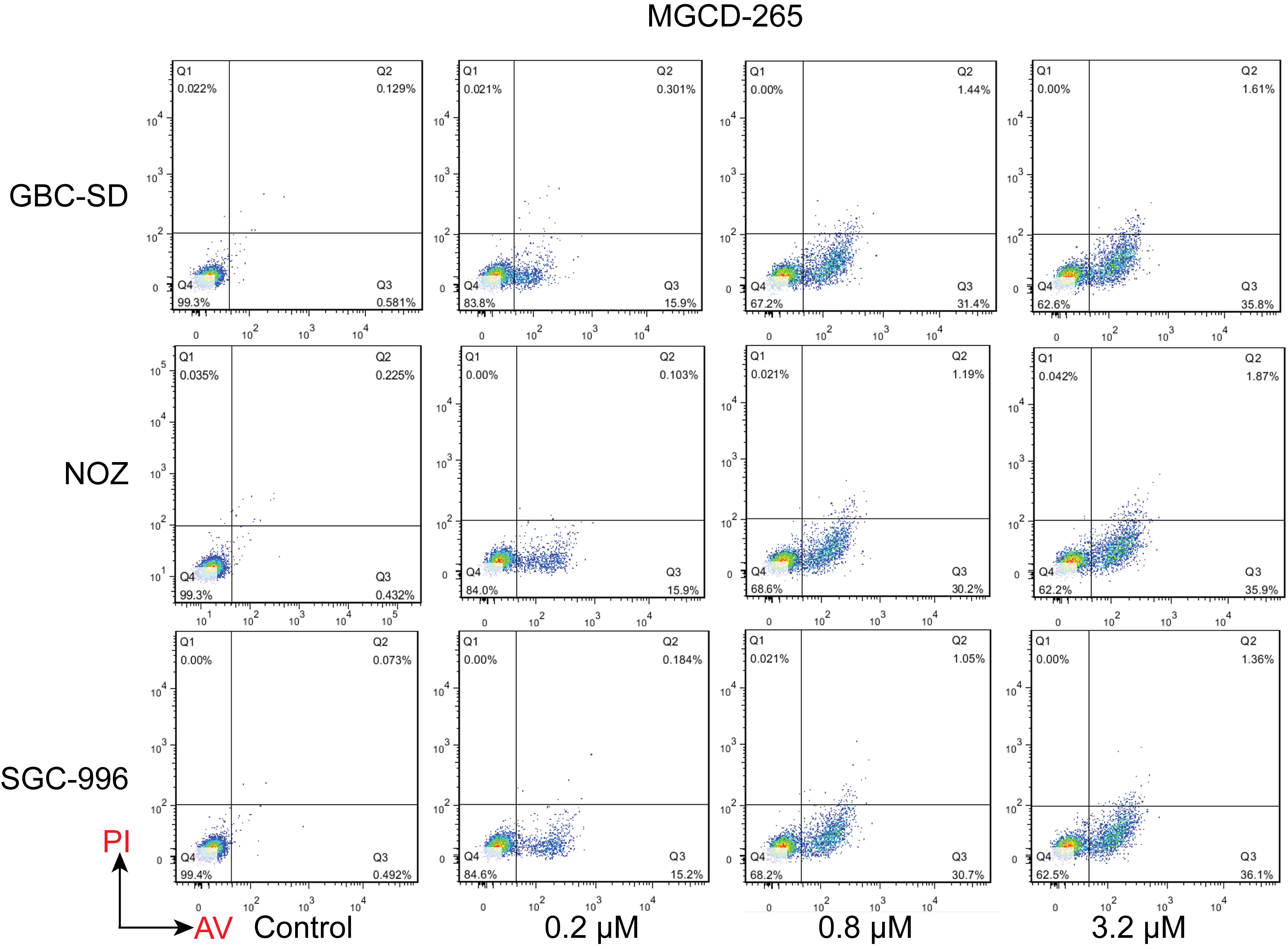

Supplement: Supplementary file 9 — Supplementary Material 9 [file 13578_2024_1290_MOESM9_ESM.tif]

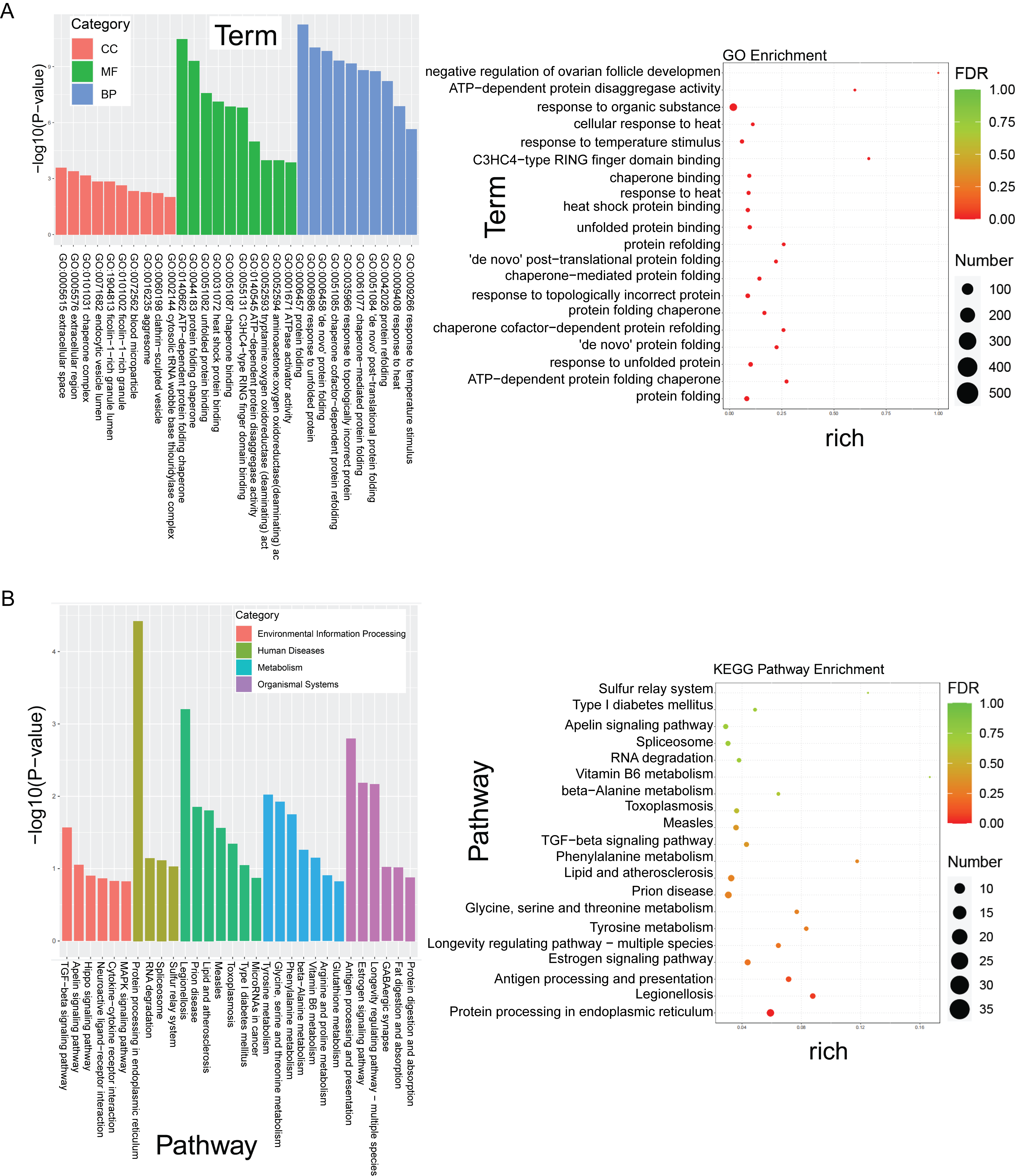

Supplement: Supplementary file 10 — Supplementary Material 10 [file 13578_2024_1290_MOESM10_ESM.tif]

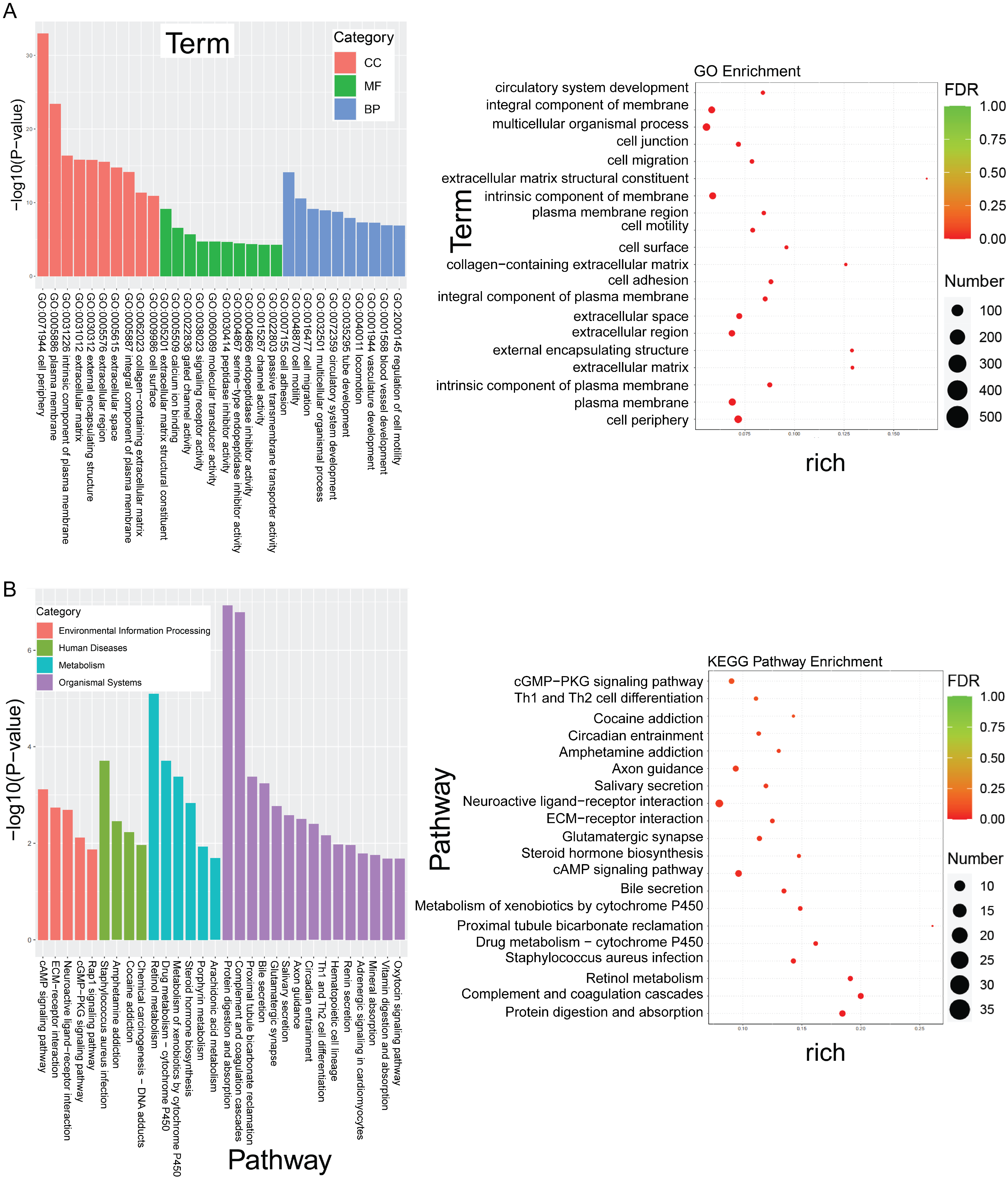

Supplement: Supplementary file 11 — Supplementary Material 11 [file 13578_2024_1290_MOESM11_ESM.tif]

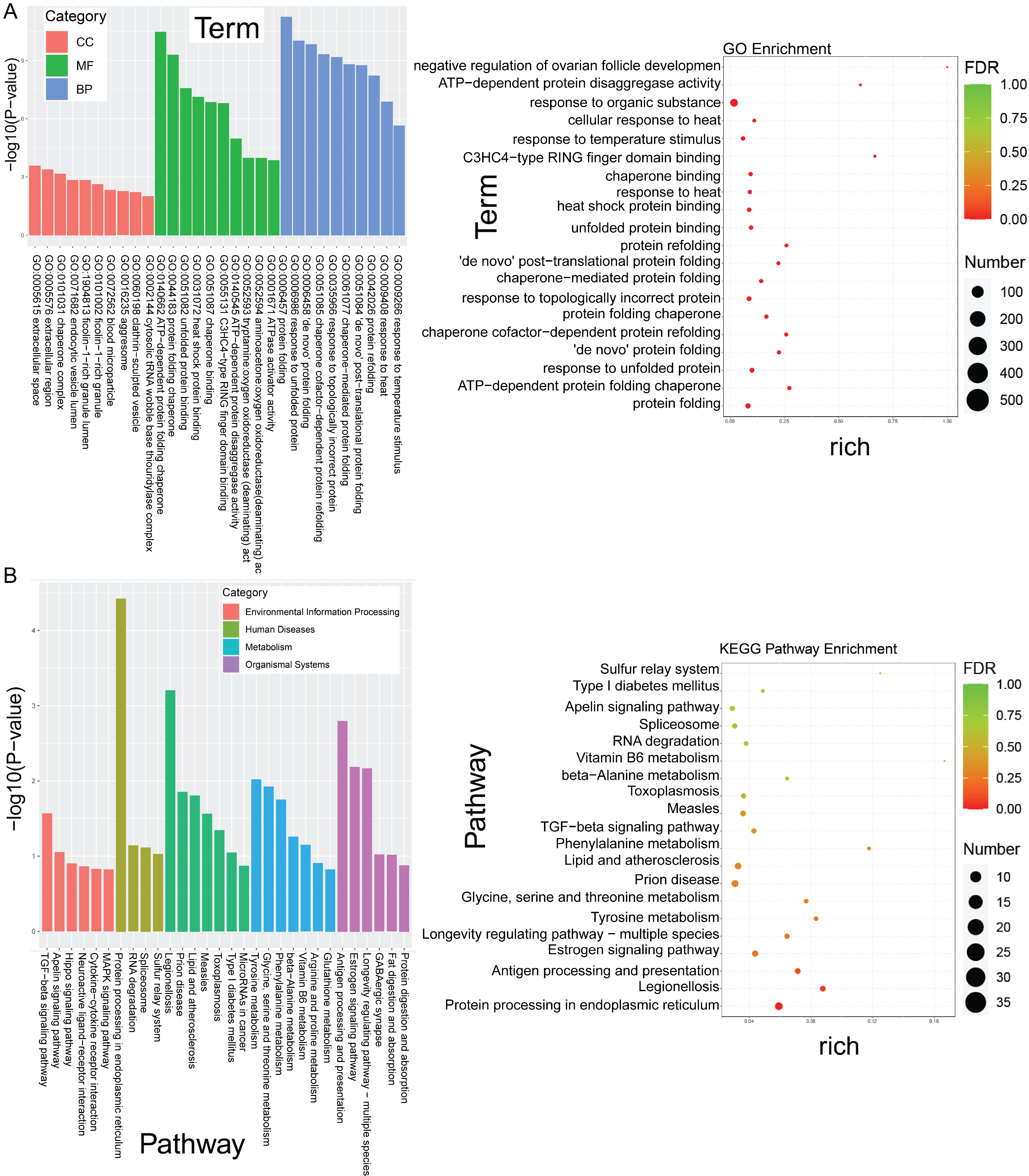

Supplement: Supplementary file 12 — Supplementary Material 12 [file 13578_2024_1290_MOESM12_ESM.tif]

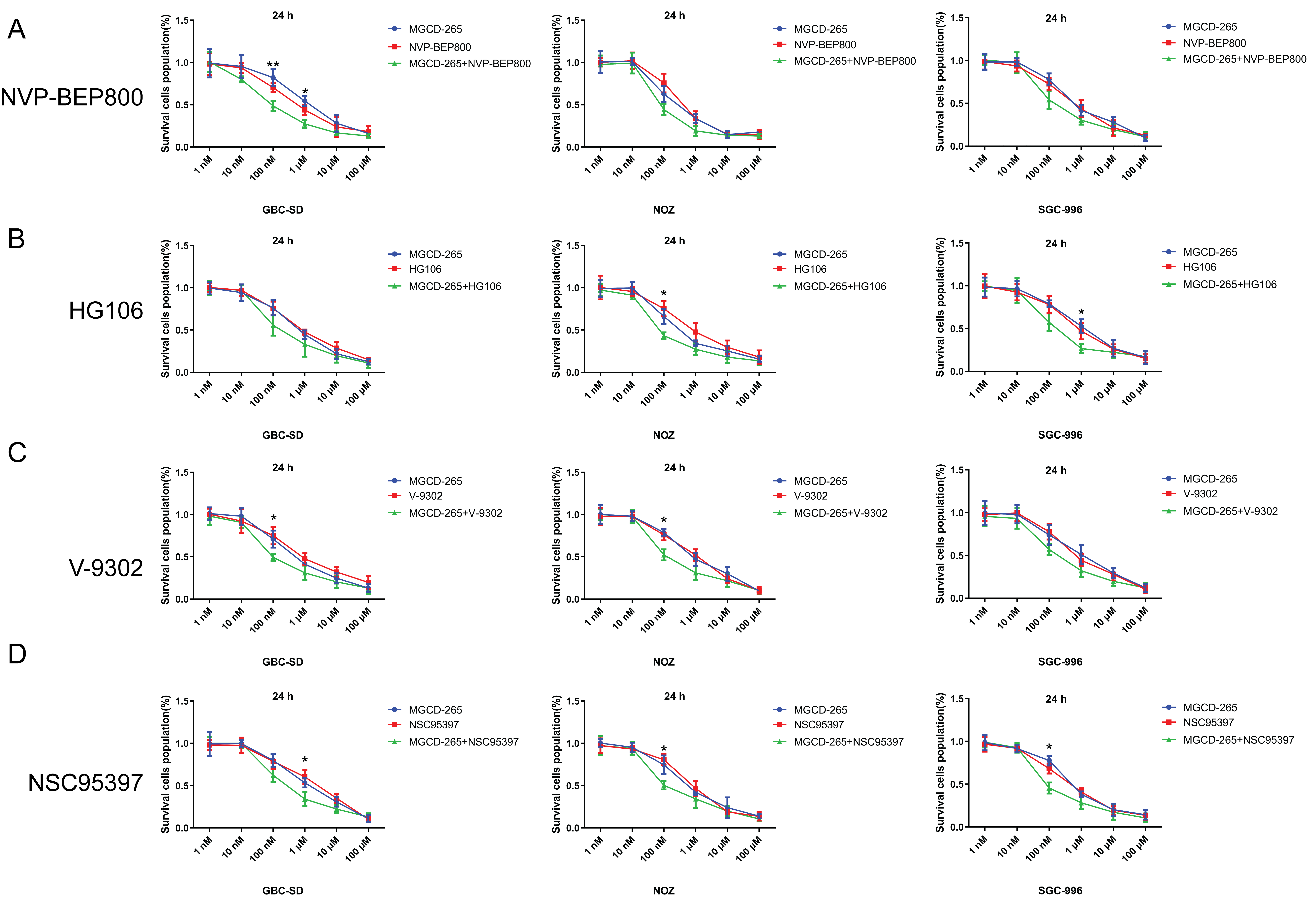

Supplement: Supplementary file 13 — Supplementary Material 13 [file 13578_2024_1290_MOESM13_ESM.tif]
