# Supplementary material for: MST1R-targeted therapy in the battle against gallbladder cancer
Source: Cell Biosci. 2024 Aug 29;14:109. doi: 10.1186/s13578-024-01290-w (PMC11363441; doi:10.1186/s13578-024-01290-w)
Supplement: Supplementary file 14 — Supplementary Material 14 [file 13578_2024_1290_MOESM14_ESM.docx]

**Supplementary figure legends**

**Figure S1** PPI analysis of differentially expressed genes in gallbladder cancer.

**Figure S2** Bioinformatics analysis to identify significantly different genes and their functions in gallbladder cancer. (A) KEGG analysis of upregulated genes in gallbladder cancer tissues. (B) GO analysis of upregulated genes in gallbladder cancer tissues. (C) KEGG analysis of downregulated genes in gallbladder cancer tissues. (D) GO analysis of downregulated genes in gallbladder cancer tissues.

**Figure S3** CCK8 assay to assess the effect of inhibitors on the proliferative capacity of gallbladder cancer GBC-SD cells.

**Figure S4** CCK8 assay to measure the effect of inhibitors on the proliferative capacity of gallbladder cancer NOZ cells.

**Figure S5** CCK8 assay to test the effect of inhibitors on the proliferative capacity of gallbladder cancer SGC-996 cells.

**Figure S6** Effects of MST1R knockdown and overexpression on GBC-SD cell proliferation and apoptosis. (A) Knockdown efficiency. (B) Cell viability of MST1R-knockdown GBC-SD cells. (C) Apoptosis of MST1R-knockdown GBC-SD cells. (A) Overexpression efficiency. (B) Cell viability of MST1R-overexpression GBC-SD cells. (C) Apoptosis of MST1R- overexpression GBC-SD cells. **p<0.01, ***p<0.001.

**Figure S7** Flow cytometric analysis showing the effect of MGCD-265 at concentrations of 0.2 μM, 0.8 μM, and 3.2 μM on cell cycle distribution of gallbladder cancer cells (GBC-SD, NOZ, SGC-996).

**Figure S8** Flow cytometric analysis showing the effect of MGCD-265 at concentrations of 0.2 μM, 0.8 μM, and 3.2 μM on apoptosis in gallbladder cancer cells (GBC-SD, NOZ, SGC-996).

**Figure S9** GO and KEGG analysis. (A) Bar chart and bubble chart representing GO enrichment analysis about differential expressed genes in gallbladder cancer GBC-SD cells treated with 0.8 μM MGCD-265 for 24 h. (B) Bar chart and bubble chart showing KEGG enrichment analysis about differential expressed genes in gallbladder cancer GBC-SD cells treated with 0.8 μM MGCD-265 for 24 h.

**Figure S10** GO and KEGG analysis of downregulated genes in MGCD-265-treated gallbladder cancer cells. (A) Bar chart and bubble chart of GO enrichment analysis for downregulated genes in gallbladder cancer GBC-SD cells treated with 0.8 μM MGCD-265 for 24 h. (B) Bar chart and bubble chart of KEGG enrichment analysis for downregulated genes in gallbladder cancer GBC-SD cells treated with 0.8 μM MGCD-265 for 24 h.

**Figure S11** GO and KEGG analysis of upregulated genes in MGCD-265-treated gallbladder cancer cells. (A) Bar chart and bubble chart of GO enrichment analysis for upregulated genes in gallbladder cancer GBC-SD cells after treatment with 0.8 μM MGCD-265 for 24 h. (B) Bar chart and bubble chart of KEGG enrichment analysis for upregulated genes in gallbladder cancer GBC-SD cells after treatment with 0.8 μM MGCD-265 for 24 h.

**Figure S12** Effect of MST1R inhibitor MGCD-265 in combination with other 4 inhibitors on proliferative ability of gallbladder cancer cells GBC-SD, NOZ, SGC-996. (A) The effect of MGCD-265 in combination with NVP-BEP800 on the proliferative ability of gallbladder cancer GBC-SD, NOZ, and SGC-996 cells. (B) The effect of MGCD-265 in combination with HG106 on the proliferative ability of gallbladder cancer cells. (C) The effect of MGCD-265 in combination with V-9302 on the proliferative ability of gallbladder cancer cells. (D) The effect of MGCD-265 in combination with NSC95397 on the proliferative ability of gallbladder cancer cells. *p<0.05, **p<0.01.

**Figure S13** Protein levels of related molecules of NF-κB and β-catenin signaling pathways in GBC-SD cells that were subjected to 3.2 μM MGCD-265 for 24 h.
